# Supplementary material for: A theoretical framework for Landsat data modeling based on the matrix variate mean-mixture of normal model
Source: PLoS One. 2020 Apr 9;15(4):e0230773. doi: 10.1371/journal.pone.0230773 (PMC7144982; doi:10.1371/journal.pone.0230773)
Supplement: Attachment — Submitted filename: A theoretical framework for Landsat data modeling based on the matrix variate meanmixture of normal model Cai.pdf [file pone.0230773.s001.pdf]

PLOS ONE

A theoretical framework for Landsat data modeling based on the matrix variate mean-mixture of normal model

--Manuscript Draft--

**Manuscript Number:**	PONE-D-19-24001
**Article Type:**	Research Article
**Full Title:**	A theoretical framework for Landsat data modeling based on the matrix variate mean-mixture of normal model
**Short Title:**	Mean-mixture of normal matrix variate model
**Corresponding Author:**	Mehrdad Naderi<br>University of Pretoria<br>Pretoria, SOUTH AFRICA
**Keywords:**	EM-type algorithm; Matrix variate distribution; Mean mixture of normal distribution; Restricted skew-normal distribution
**Abstract:**	This paper introduces a new family of matrix variate distributions based on the mean-mixture<br>of normal (MMN) model. The properties of the new matrix variate family, namely stochastic<br>representation, moments and characteristic function, linear and quadratic forms as well as marginal<br>and conditional distributions are investigated. Three special cases including the restricted skew-normal,<br>exponentiated MMN and the mixed-Weibull MMN matrix variate distributions are presented and<br>studied. Based on the specific presentation of the proposed model, an EM-type algorithm can be<br>directly implemented for obtaining maximum likelihood estimate of the parameters. The usefulness<br>and practical utility of the proposed methodology are illustrated through two conducted simulation<br>studies and through the landsat satellite dataset analysis.
**Order of Authors:**	Mehrdad Naderi
	Andriette Bekker
	Mohammad Arashi
	Ahad Jamalizadeh
**Additional Information:**	
**Question**	**Response**
**Financial Disclosure**<br><br>Enter a financial disclosure statement that describes the sources of funding for the work included in this submission. Review the submission guidelines for detailed requirements. View published research articles from *PLOS ONE* for specific examples.<br><br>This statement is required for submission and **will appear in the published article** if the submission is accepted. Please make	M. Naderi, A. Bekker and M. Arashi received the National Research Foundation (NRF) of South Africa, support<br>Reference: CPRR160403161466 grant Number: 105840 and STATOMET and<br>Ref: IFR170227223754 grant Number: 109214 and SARChI Research Chair-UID: 71199.

Powered by Editorial Manager® and ProduXion Manager® from Aries Systems Corporation

sure it is accurate.<br><br>**Unfunded studies**<br>Enter: *The author(s) received no specific funding for this work.*<br><br>**Funded studies**<br>Enter a statement with the following details:<br>• Initials of the authors who received each award<br>• Grant numbers awarded to each author<br>• The full name of each funder<br>• URL of each funder website<br>• Did the sponsors or funders play any role in the study design, data collection and analysis, decision to publish, or preparation of the manuscript?<br>• **NO** - Include this sentence at the end of your statement: *The funders had no role in study design, data collection and analysis, decision to publish, or preparation of the manuscript.*<br>• **YES** - Specify the role(s) played.<br><br>* typeset	
**Competing Interests**<br><br>Use the instructions below to enter a competing interest statement for this submission. On behalf of all authors, disclose any competing interests that could be perceived to bias this work—acknowledging all financial support and any other relevant financial or non-financial competing interests.<br><br>This statement **will appear in the published article** if the submission is accepted. Please make sure it is accurate. View published research articles from *PLOS ONE* for specific examples.	NO

Powered by Editorial Manager® and ProduXion Manager® from Aries Systems Corporation

**NO authors have competing interests**<br><br>Enter: *The authors have declared that no competing interests exist.*<br><br>**Authors with competing interests**<br><br>Enter competing interest details beginning with this statement:<br><br>*I have read the journal's policy and the authors of this manuscript have the following competing interests: [insert competing interests here]*<br><br>* typeset	
**Ethics Statement**<br><br>Enter an ethics statement for this submission. This statement is required if the study involved:<br><br>• Human participants<br>• Human specimens or tissue<br>• Vertebrate animals or cephalopods<br>• Vertebrate embryos or tissues<br>• Field research<br><br>Write "N/A" if the submission does not require an ethics statement.<br><br>General guidance is provided below. Consult the submission guidelines for detailed instructions. **Make sure that all information entered here is included in the Methods section of the manuscript.**	N/A

Powered by Editorial Manager® and ProduXion Manager® from Aries Systems Corporation

**Format for specific study types**<br><br>**Human Subject Research (involving human participants and/or tissue)**<br>• Give the name of the institutional review board or ethics committee that approved the study<br>• Include the approval number and/or a statement indicating approval of this research<br>• Indicate the form of consent obtained (written/oral) or the reason that consent was not obtained (e.g. the data were analyzed anonymously)<br><br>**Animal Research (involving vertebrate animals, embryos or tissues)**<br>• Provide the name of the Institutional Animal Care and Use Committee (IACUC) or other relevant ethics board that reviewed the study protocol, and indicate whether they approved this research or granted a formal waiver of ethical approval<br>• Include an approval number if one was obtained<br>• If the study involved *non-human primates*, add *additional details* about animal welfare and steps taken to ameliorate suffering<br>• If anesthesia, euthanasia, or any kind of animal sacrifice is part of the study, include briefly which substances and/or methods were applied<br><br>**Field Research**<br><br>Include the following details if this study involves the collection of plant, animal, or other materials from a natural setting:<br>• Field permit number<br>• Name of the institution or relevant body that granted permission	
**Data Availability**<br><br>Authors are required to make all data underlying the findings described fully available, without restriction, and from the time of publication. PLOS allows rare exceptions to address legal and ethical concerns. See the PLOS Data Policy and FAQ for detailed information.	Yes - all data are fully available without restriction

Powered by Editorial Manager® and ProduXion Manager® from Aries Systems Corporation

A Data Availability Statement describing where the data can be found is required at submission. Your answers to this question constitute the Data Availability Statement and **will be published in the article**, if accepted.<br><br>**Important:** Stating 'data available on request from the author' is not sufficient. If your data are only available upon request, select 'No' for the first question and explain your exceptional situation in the text box.<br><br>Do the authors confirm that all data underlying the findings described in their manuscript are fully available without restriction?	
**Describe where the data may be found in full sentences. If you are copying our sample text, replace any instances of XXX with the appropriate details.**<br><br>• If the data are **held or will be held in a public repository**, include URLs, accession numbers or DOIs. If this information will only be available after acceptance, indicate this by ticking the box below. For example: *All XXX files are available from the XXX database (accession number(s) XXX, XXX.).*<br>• If the data are all contained **within the manuscript and/or Supporting Information files**, enter the following: *All relevant data are within the manuscript and its Supporting Information files.*<br>• If neither of these applies but you are able to provide **details of access elsewhere**, with or without limitations, please do so. For example:<br><br>*Data cannot be shared publicly because of [XXX]. Data are available from the XXX Institutional Data Access / Ethics Committee (contact via XXX) for researchers who meet the criteria for access to confidential data.*<br><br>*The data underlying the results presented in the study are available from (include the name of the third party*	http://archive.ics.uci.edu/ml

and contact information or URL).<br>• This text is appropriate if the data are owned by a third party and authors do not have permission to share the data.<br><br>* typeset	
Additional data availability information:	

Powered by Editorial Manager® and ProduXion Manager® from Aries Systems Corporation

Click here to download Manuscript plos_latex_template.pdf

A theoretical framework for Landsat data modeling based on the matrix variate mean-mixture of normal model

Mehrdad Naderi[1], Andriette Bekker[1], Mohammad Arashi[1,2], Ahad Jamalizadeh[3]

1 Department of Statistics, Faculty of Natural & Agricultural Sciences, University of Pretoria, Pretoria, South Africa
2 Department of Statistics, School of Mathematical Sciences, Shahrood University of Technology, Shahrood, Iran
3 Department of Statistics, Faculty of Mathematics and Computer, Shahid Bahonar University of Kerman, Kerman, Iran

Abstract

This paper introduces a new family of matrix variate distributions based on the mean-mixture of normal (MMN) model. The properties of the new matrix variate family, namely stochastic representation, moments and characteristic function, linear and quadratic forms as well as marginal and conditional distributions are investigated. Three special cases including the restricted skew-normal, exponentiated MMN and the mixed-Weibull MMN matrix variate distributions are presented and studied. Based on the specific presentation of the proposed model, an EM-type algorithm can be directly implemented for obtaining maximum likelihood estimate of the parameters. The usefulness and practical utility of the proposed methodology are illustrated through two conducted simulation studies and through the landsat satellite dataset analysis.

1 Introduction

The skew-normal (SN) distribution, initially introduced by Azzalini [1], has received considerable attention in both theoretical and applied statistics in the past two decades. Various extensions, forms and properties of the SN distribution in the multivariate case were derived in [2–5], and the acknowledged articles therein. An interesting form of the SN distribution was presented by Pyne et al. [3] who named it the restricted multivariate SN (rSN) model. Generally, the rSN distribution can be expressed as a linear transformation of the multivariate normally distributed random vector and the univariate truncated normal distribution. Although the rSN model, like the original SN one, can describe the skewness of data, it still is not robust in dealing with the outlying observations. To cover this drawback, Negarestani et al. [6] used the rSN transformation to introduce the family of multivariate mean mixture of normal (MMN) model. Specifically, a p-dimension random vector $\boldsymbol{X}$ is in the family of MMN distributions if

$$\boldsymbol{X} \stackrel{d}{=} \boldsymbol{\mu} + \boldsymbol{\lambda} W + \boldsymbol{Z}, \tag{1}$$

where '$\stackrel{d}{=}$' stands for the equality in distribution, $\boldsymbol{Z}$ follows the multivariate normal model and W is an arbitrary random variable independent of $\boldsymbol{Z}$. It is clear that the rSN distribution is a special case of (1) where the mixing variable W is followed by the truncated standard normal distribution lying within a truncated interval $(0, \infty)$,

denoted by $W \sim \mathcal{TN}(0,1;(0,\infty))$. It is shown by Negarestani et al. [6] that the family of MMN may provide a new model with wider range of skewness and kurtosis than the rSN, skew-t [4] and skew Student-t-normal [7] distributions. From (1), the probability distribution function (pdf) of random vector $\boldsymbol{X}$ can be presented as

$$f_{\mathrm{MMN}_p}(\boldsymbol{x};\boldsymbol{\mu},\boldsymbol{\lambda},\boldsymbol{\Sigma},\boldsymbol{\nu}) = \int_{-\infty}^{\infty} \phi(\boldsymbol{x};\boldsymbol{\mu}+\boldsymbol{\lambda}w,\boldsymbol{\Sigma})h(w;\boldsymbol{\nu})\,dw, \quad \boldsymbol{x} \in \mathbb{R}^p, \tag{2}$$

where $h(\cdot;\boldsymbol{\nu})$ denotes the pdf of W parameterized by the vector parameter $\boldsymbol{\nu}$. The notation $\boldsymbol{X} \sim \mathcal{MMN}_p(\boldsymbol{\mu},\boldsymbol{\lambda},\boldsymbol{\Sigma},W)$ will be used to indicate that $\boldsymbol{X}$ has pdf (2). Depending on the random variable W that can take values on the real line, the pdf (2) can be both symmetric and asymmetric. However, a more flexible and skewed version of the MMN model can be obtain if W has any asymmetric distribution or any positive support model like the truncated-normal, exponential and gamma distributions. Moreover, the pdf (2) can include skew-elliptical models, as the rSN distribution, or can result in skew non-elliptically contoured models if, for example, W is distributed as the exponential, Weibull and gamma models. From Figure 2 in Appendix A, it is observed that the family of MMN distributions offers different orientation compared with the family of mean-variance mixture of normal (MVMN) distributions [8].

Matrix variate distribution finds its genesis in modeling dependent multivariate observations in the normal case [9]. The recent use of the matrix variate normal (MVN) distribution can be found in modeling a wide variety of three-way data appearing in studies including control theory, stochastic systems, image recognition, repeated vector measurements, multivariate time series, spatial data, among others [10, 11]. The MVN distribution not only inherits some appealing properties, features as well as widespread applications from the multivariate normal model, but also it is still not stable and robust against non-normal features such as asymmetry and heavy tails. To deal with the heavy tailed data, Kshirsagar and Bartlett [12] proposed the matrix variate t distribution by showing that the estimator of the parameter matrix of regression coefficients unconditionally follows matrix variate t model. Bulut and Arslan [13] proposed the matrix variate slash distribution as a scale mixture of the matrix variate normal and the uniform distributions. Moreover, in accommodating skewness and kurtosis, the interest of skew distributions provides a platform for robust extension of matrix variate distribution. For instance, works on the matrix variate versions of SN distribution can be found in [14–17]. Even though the matrix variate SN distribution has many attractive properties, it suffers from robustness in dealing with heavy tailed data and from parameter estimation. Regarding these drawbacks of the matrix variate SN model and considering the aforementioned properties of the MMN family of distributions, the objective of this paper is to propose a family of matrix variate mean-mixture of normal (MVMMN) distributions. Some properties and features of our introduced family such as moments, the characteristic function, marginal and conditional distributions are studied. The maximum likelihood (ML) estimate of model parameters are computed by applying expectation-maximization (EM) type algorithm [18].

The contribution of this work can be broken down into six parts. We will begin the usual procedure with the model formulation of the MVMMN distribution in Section 2. Properties and characteristics of the MVMMN distribution are also studied in Section 3. The parameter estimation procedure using the EM-type algorithm and some computational strategies of implementation are given in Section 4. To examine the performance of the methodology into practice, simulation and real-world data analyses are presented in Sections 5 and 6. Finally, Section 7 gives some concluding remarks and future extensions.

2 Proposed family

To start the whole process, we begin with the definition of the MVMMN model.

Definition 1 *A random matrix* $\boldsymbol{\mathcal{Y}}$ *is said to have a MVMMN distribution if it can be generated by the following stochastic representation*

$$\boldsymbol{\mathcal{Y}} \overset{d}{=} \boldsymbol{M} + W\boldsymbol{\Lambda} + \boldsymbol{\mathcal{X}}, \tag{3}$$

where $\boldsymbol{M}, \boldsymbol{\Lambda} \in \mathbb{R}^{p\times n}$ *is a skewness matrix,* $\boldsymbol{\mathcal{X}} \in \mathbb{R}^{p\times n}$ *follows a MVN distribution with mean matrix* $\mathbf{0}$ *and dispersion matrices* $\boldsymbol{\Sigma} \in \mathbb{R}^{p\times p}$ *and* $\boldsymbol{\Psi} \in \mathbb{R}^{n\times n}$*, denoted by* $\boldsymbol{\mathcal{X}} \sim \mathcal{N}_{p,n}(\mathbf{0}, \boldsymbol{\Sigma}, \boldsymbol{\Psi})$*, and independently of* $\boldsymbol{\mathcal{X}}$*,* W *is a random variable distributed by* $h(w;\boldsymbol{\nu})$*. It can be easily seen that the hierarchical representation of MVMMN model is*

$$\begin{aligned} \boldsymbol{\mathcal{Y}}|W = w &\sim \mathcal{N}_{p,n}(\boldsymbol{M} + w\boldsymbol{\Lambda}, \boldsymbol{\Sigma}, \boldsymbol{\Psi}), \\ W &\sim h(w;\boldsymbol{\nu}). \end{aligned} \tag{4}$$

Hence, the pdf of $\boldsymbol{\mathcal{Y}} \sim \mathcal{MVMMN}_{p,n}(\boldsymbol{M}, \boldsymbol{\Lambda}, \boldsymbol{\Sigma}, \boldsymbol{\Psi}, W)$ *can be given as*

$$f(\boldsymbol{Y}; \boldsymbol{M}, \boldsymbol{\Lambda}, \boldsymbol{\Sigma}, \boldsymbol{\Psi}, \boldsymbol{\nu}) = \int_{-\infty}^{\infty} \phi_{p,n}(\boldsymbol{Y}; \boldsymbol{M} + w\boldsymbol{\Lambda}, \boldsymbol{\Sigma}, \boldsymbol{\Psi}) h(w;\boldsymbol{\nu})\, dw, \qquad \boldsymbol{Y} \in \mathbb{R}^{p\times n}. \tag{5}$$

where $\phi_{p,n}(\cdot; \boldsymbol{M}, \boldsymbol{\Sigma}, \boldsymbol{\Psi})$ *is the pdf of* $\boldsymbol{\mathcal{X}} \sim \mathcal{N}_{p,n}(\boldsymbol{M}, \boldsymbol{\Sigma}, \boldsymbol{\Psi})$*.*

Applying the well-known property of the MVN distribution, we have

$$\boldsymbol{\mathcal{Y}} \sim \mathcal{MVMMN}_{p,n}(\mathbf{M}, \boldsymbol{\Lambda}, \boldsymbol{\Sigma}, \boldsymbol{\Psi}, W) \Longleftrightarrow \mathrm{vec}(\boldsymbol{\mathcal{Y}}) \sim \mathcal{MMN}_{pn}(\mathrm{vec}(\mathbf{M}), \mathrm{vec}(\boldsymbol{\Lambda}), \boldsymbol{\Psi}\otimes\boldsymbol{\Sigma}, W), \tag{6}$$

where $\mathrm{vec}(B)$ denotes the vectorization operator of matrix B, and $\otimes$ stands for the Kronecker product.

Remark 1 *It is obvious that the MVMMN distribution assumes that the mean is not fixed for all members of the population. We would like to emphasize that the family of matrix variate normal mean-variance mixture (MVNMVM) models [19, 20], assumes that both the mean and variance of the population member are not fixed. Therefore, an interesting extension of the MVMMN distribution can be introduced by considering the family of scale mixture of MVMMN distributions.*

2.1 Special cases

- **Restricted matrix variable skew-normal:** If $W \sim \mathcal{TN}(0, 1; (0, \infty))$ in (3), then restricted matrix variable SN (RMVSN) distribution is arisen. The resulting pdf of $\boldsymbol{\mathcal{Y}}$ directly obtained by integrating out (5), is

$$f_{\text{RMVSN}}(\mathbf{Y}; \mathbf{M}, \boldsymbol{\Lambda}, \boldsymbol{\Sigma}, \boldsymbol{\Psi}) = \frac{2}{\boldsymbol{\eta}} \exp\left\{\frac{\mathbf{A}^2}{2}\right\} \phi_{p,n}(\mathbf{Y}; \mathbf{M}, \boldsymbol{\Sigma}, \boldsymbol{\Psi}) \Phi(\mathbf{A}), \qquad \mathbf{Y} \in \mathbb{R}^{p\times n},$$

where $\boldsymbol{\eta}^2 = \mathrm{tr}\left(\boldsymbol{\Psi}^{-1}\boldsymbol{\Lambda}^\top\boldsymbol{\Sigma}^{-1}\boldsymbol{\Lambda}\right) + 1$, $\mathbf{A} = \boldsymbol{\eta}^{-1}\left[\mathrm{tr}\left(\boldsymbol{\Psi}^{-1}\boldsymbol{\Lambda}^\top\boldsymbol{\Sigma}^{-1}(\mathbf{Y} - \mathbf{M})\right)\right]$, and $\Phi(\cdot)$ denotes the cumulative distribution function (CDF) of standard normal model and $\mathrm{tr}(\cdot)$ denotes the trace operator.

Lemma 1 *If* $W \sim \mathcal{TN}\left(\mu, \sigma^2; (0, \infty)\right)$*, then*

$$E(W) = \mu + \sigma\frac{\phi(\mu/\sigma)}{\Phi(\mu/\sigma)}, \qquad E(W^r) = \mu E\left(W^{r-1}\right) + (r-1)\sigma^2 E\left(W^{r-2}\right), \quad r = 2, 3, \ldots.$$

Proposition 1 *Let* $\boldsymbol{\mathcal{Y}} \sim \mathcal{RMVSN}(\boldsymbol{M}, \boldsymbol{\Lambda}, \boldsymbol{\Sigma}, \boldsymbol{\Psi})$ *and* $W \sim \mathcal{TN}(0, 1; (0, \infty))$*. Then, W conditionally on* $\boldsymbol{\mathcal{Y}} = \boldsymbol{Y}$*, denoted by* $W_{\boldsymbol{Y}}$*, follows* $\mathcal{TN}\left(\boldsymbol{A}/\boldsymbol{\eta}, 1/\boldsymbol{\eta}^2; (0, \infty)\right)$.

Proof. The proof follows from Bayes' rule and some matrix factorizations. Using hierarchical representation (4) and the pdf of RMVSN model, we have

$$\begin{aligned} f_{W_{\mathbf{Y}}}(w; \mathbf{M}, \boldsymbol{\Lambda}, \boldsymbol{\Sigma}, \boldsymbol{\Psi}) &= \frac{2\phi_{p,n}(\mathbf{Y}; \mathbf{M} - w\boldsymbol{\Lambda}, \boldsymbol{\Sigma}, \boldsymbol{\Psi})\phi(w)}{f_{\text{RMVSN}}(\mathbf{Y}; \mathbf{M}, \boldsymbol{\Lambda}, \boldsymbol{\Sigma}, \boldsymbol{\Psi})} \\ &= \frac{\boldsymbol{\eta} \exp\left\{-0.5\left(w^2\left[\text{tr}\left(\boldsymbol{\Sigma}^{-1}\boldsymbol{\Lambda}\boldsymbol{\Psi}^{-1}\boldsymbol{\Lambda}^\top\right) + 1\right] - 2w \ \text{tr}\left(\boldsymbol{\Sigma}^{-1}\boldsymbol{\Lambda}\boldsymbol{\Psi}^{-1}(\mathbf{Y} - \mathbf{M})^\top\right) + \mathbf{A}^2\right)\right\}}{\sqrt{2\pi} \ \Phi(\mathbf{A})} \\ &= \frac{\phi(w; \mathbf{A}/\boldsymbol{\eta}, 1/\boldsymbol{\eta}^2)}{\Phi(\mathbf{A})}, \end{aligned}$$

which completes the proof.

• **Convolution with exponential model:** The exponentiated MVMMN (MVMMNE) distribution, say $\boldsymbol{\mathcal{Y}} \sim \mathcal{MVMMNE}(\mathbf{M}, \boldsymbol{\Lambda}, \boldsymbol{\Sigma}, \boldsymbol{\Psi})$, is derived as another especial case of (3) if $W \sim \mathcal{E}(1)$, where $\mathcal{E}(1)$ denotes the exponential distribution with mean 1. This leads to obtain the pdf of $\boldsymbol{\mathcal{Y}}$ form (5) as

$$f_{\text{MVMMNE}}(\mathbf{Y}; \mathbf{M}, \boldsymbol{\Lambda}, \boldsymbol{\Sigma}, \boldsymbol{\Psi}) = \frac{\sqrt{2\pi}}{\boldsymbol{\eta}_1^*} \exp\left\{\frac{\mathbf{A}_1^{*2}}{2}\right\} \phi_{p,n}(\mathbf{Y}; \mathbf{M}, \boldsymbol{\Sigma}, \boldsymbol{\Psi})\Phi(\mathbf{A}_1^*), \qquad \mathbf{Y} \in \mathbb{R}^{p\times n},$$

where $\boldsymbol{\eta}_1^{*2} = \boldsymbol{\eta}^2 - 1$, $\mathbf{A}_1^* = \left[\text{tr}\left(\boldsymbol{\Psi}^{-1}\boldsymbol{\Lambda}^\top\boldsymbol{\Sigma}^{-1}\left(\mathbf{Y} - \mathbf{M}\right)\right) - 1\right]/\boldsymbol{\eta}_1^*$.

Proposition 2 *Let* $\boldsymbol{\mathcal{Y}} \sim \mathcal{MVMMNE}(\boldsymbol{M}, \boldsymbol{\Lambda}, \boldsymbol{\Sigma}, \boldsymbol{\Psi})$ *and* $W \sim \mathcal{E}(1)$*. Then,* $W_{\boldsymbol{Y}} \sim \mathcal{TN}\left(\boldsymbol{A}_1^*/\boldsymbol{\eta}_1^*, 1/\boldsymbol{\eta}_1^{*2}; (0, \infty)\right)$.

Proof. Using Bayes' rule and some matrix factorizations, the proof is completed.

• **Convolution with Weibull model:** The mixed-Weibull MVMMN (MVMMNW) distribution, denoted by $\boldsymbol{\mathcal{Y}} \sim \mathcal{MVMMNW}(\mathbf{M}, \boldsymbol{\Lambda}, \boldsymbol{\Sigma}, \boldsymbol{\Psi})$, is arisen when W in (3) follows the Weibull model respectively with shape and scale parameters $\alpha = 2$ and $\beta = 1$, $\mathcal{WE}(2, 1)$. Hence, the associated pdf of $\boldsymbol{\mathcal{Y}} \sim \mathcal{MVMMNW}(\mathbf{M}, \boldsymbol{\Lambda}, \boldsymbol{\Sigma}, \boldsymbol{\Psi})$ obtained by (5) is

$$\begin{aligned} f_{\text{MVMMW}}(\mathbf{Y}; \mathbf{M}, \boldsymbol{\Lambda}, \boldsymbol{\Sigma}, \boldsymbol{\Psi}) =& \frac{2\sqrt{2\pi}}{\boldsymbol{\eta}_2^{*2}} \exp\left\{\frac{\mathbf{A}_2^{*2}}{2}\right\} \phi_{p,n}(\mathbf{Y}; \mathbf{M}, \boldsymbol{\Sigma}, \boldsymbol{\Psi}) \\ &\times \left(\mathbf{A}_2^*\Phi(\mathbf{A}_2^*) + \phi(\mathbf{A}_2^*)\right), \quad \mathbf{Y} \in \mathbb{R}^{p\times n}, \end{aligned}$$

where $\boldsymbol{\eta}_2^{*2} = \boldsymbol{\eta}^2 + 1$, $\mathbf{A}_2^* = \text{tr}\left(\boldsymbol{\Psi}^{-1}\boldsymbol{\Lambda}^\top\boldsymbol{\Sigma}^{-1}\left(\mathbf{Y} - \mathbf{M}\right)\right)/\boldsymbol{\eta}_2^*$.

Proposition 3 *Let* $\boldsymbol{\mathcal{Y}} \sim \mathcal{MVMMNW}(\boldsymbol{M}, \boldsymbol{\Lambda}, \boldsymbol{\Sigma}, \boldsymbol{\Psi})$ *and* $W \sim \mathcal{WE}(2, 1)$*. Then,* $W_{\boldsymbol{Y}}$ *has the pdf*

$$f_{W_{\boldsymbol{Y}}}(w; \boldsymbol{M}, \boldsymbol{\Lambda}, \boldsymbol{\Sigma}, \boldsymbol{\Psi}) = \frac{w \ \eta_2^*}{\boldsymbol{A}_2^*\Phi(\boldsymbol{A}_2^*) + \phi(\boldsymbol{A}_2^*)} \phi(w; \boldsymbol{A}_2^*/\boldsymbol{\eta}_2^*, 1/\boldsymbol{\eta}_2^{*2}).$$

Moreover, for $r = 1, 2, \ldots,$

$$E\left(W^r \middle| \boldsymbol{\mathcal{Y}} = \boldsymbol{Y}\right) = \frac{\eta_2^*\Phi(\boldsymbol{A}_2^*)}{\boldsymbol{A}_2^*\Phi(\boldsymbol{A}_2^*) + \phi(\boldsymbol{A}_2^*)} E\left(V^{r+1}\right).$$

where $V \sim \mathcal{TN}\left(\boldsymbol{A}_2^*/\boldsymbol{\eta}_2^*, 1/\boldsymbol{\eta}_2^{*2}; (0, \infty)\right)$.

Proof. Results can be obtained from Bayes' rule and some matrix factorizations.

Theorem 1 *The MVMMN distribution is log-concave if W has log-concave pdf.*

Proof. Based on [21], if $f(x)$ and $g(y)$ are log-concave functions, then their convolution, i.e.,

$$\int h(x,y)dy = \int f(x-y)g(y)dy,$$

is also a log-concave function. Hence, the property of vectorization operator of the MVMMN distribution (6) and the fact that the MVN is log-concave completes the proof if W has a log-concave pdf.

Corollary 1 *The RMVSN, MVMMNE and MVMMNW distributions are log-concave.*

Proof. Since the truncated normal, exponential and Weibull (if the shape parameter is ≥ 1) distributions are log-concave, their associated matrix variate models are, using Theorem 1.

3 Characteristics

This section provides some substantial statistical properties of the MVMMN distribution.

Theorem 2 *If $\boldsymbol{\mathcal{Y}} \sim \mathcal{MVMMN}_{p,n}(\boldsymbol{M}, \boldsymbol{\Lambda}, \boldsymbol{\Sigma}, \boldsymbol{\Psi}, W)$, then the mean and the characteristic function of $\boldsymbol{\mathcal{Y}}$, respectively, are*

$$E(\boldsymbol{\mathcal{Y}}) = \boldsymbol{M} + E(W)\boldsymbol{\Lambda},$$
$$\varphi_{\boldsymbol{\mathcal{Y}}}(\boldsymbol{T}) = etr\left\{i\boldsymbol{T}^\top \boldsymbol{M} - \frac{1}{2}\boldsymbol{T}^\top \boldsymbol{\Sigma}\boldsymbol{T}\boldsymbol{\Psi}\right\} \varphi_W\left(tr\left(\boldsymbol{T}^\top\boldsymbol{\Lambda}\right)\right), \quad i=\sqrt{-1},$$

where $etr\{A\} = \exp(tr(A))$, $\varphi_W(\cdot)$ is the characteristic function of $W \sim h(w;\boldsymbol{\nu})$.

Proof. The proof of theorem can be completed by using the presented representations in Definition 1. Taking expectation from both side of the stochastic representation (3) leads to obtain the first part. Moreover, recall that the characteristic function of matrix variate $\boldsymbol{\mathcal{X}} \sim \mathcal{N}_{p,n}(\mathbf{M}, \boldsymbol{\Sigma}, \boldsymbol{\Psi})$ is given as

$$\varphi_{\boldsymbol{\mathcal{X}}}(\boldsymbol{T}) = \text{etr}\left\{i\boldsymbol{T}^\top \mathbf{M} - \frac{1}{2}\boldsymbol{T}^\top \boldsymbol{\Sigma}\boldsymbol{T}\boldsymbol{\Psi}\right\}$$

Hence, through the hierarchical representation (4), the characteristic function of $\boldsymbol{\mathcal{Y}}$ is obtained by $E(E(\text{tr}\left(i\boldsymbol{T}^\top\boldsymbol{\mathcal{Y}}\right)|W=w))$.

Theorem 3 *Let $\boldsymbol{\mathcal{Y}} \sim \mathcal{MVMMN}_{p,n}(\boldsymbol{M}, \boldsymbol{\Lambda}, \boldsymbol{\Sigma}, \boldsymbol{\Psi}, W)$, and $\boldsymbol{M} = (m_{ij}), \boldsymbol{\Lambda} = (\lambda_{ij}), \boldsymbol{\Sigma} = (\sigma_{ij}), \boldsymbol{\Psi} = (\psi_{ij})$. Then, we have*

(i) For $1 < i_1, i_2 < p$, and $1 < j_1, j_2 < n$,

$$E(Y_{i_1j_1}Y_{i_2j_2}) = \sigma_{i_1j_1}\sigma_{i_2j_2} + m_{i_1j_1}m_{i_2j_2} + E(W)(m_{i_1j_1}\lambda_{i_2j_2} + \lambda_{i_1j_1}m_{i_2j_2}) + E(W^2)\lambda_{i_1j_1}\lambda_{i_2j_2}.$$

(ii) If $\boldsymbol{M} = \boldsymbol{0}$,

$$E\left(tr\left(\boldsymbol{\mathcal{Y}}\boldsymbol{\mathcal{Y}}^\top\right)\right) = E(W^2)\sum_{i=1}^{p}\sum_{j=1}^{n}\lambda_{ij}^2 + tr\left(\boldsymbol{\Sigma}\right) tr\left(\boldsymbol{\Psi}\right).$$

Proof. (i) follows by using hierarchical representation (4) and applying theorems 2.3.3 of [22]. For $\mathbf{M} = \mathbf{0}$, it is clear from part (i) that

$$E(Y_{i_1j_1}Y_{i_2j_2}) = \sigma_{i_1j_1}\sigma_{i_2j_2} + E(W^2)\lambda_{i_1j_1}\lambda_{i_2j_2}.$$

Therefore, we have

$$E\left(\operatorname{tr}\left(\boldsymbol{\mathcal{Y}}\boldsymbol{\mathcal{Y}}^\top\right)\right) = E\left(\sum_{i=1}^{p}\sum_{j=1}^{n} Y_{ij}Y_{ij}\right) = \sum_{i=1}^{p}\sum_{j=1}^{n} E(Y_{ij}Y_{ij}) = \sum_{i=1}^{p}\sum_{j=1}^{n} \sigma_{ii}\psi_{jj} + E(W^2)\lambda_{ij}\lambda_{ij},$$

which completes the proof.

Theorem 4 *The family of MVMMN distributions is closed under the transpose operator, i.e.,*

$$\boldsymbol{\mathcal{Y}} \sim \mathcal{MVMMN}_{p,n}(\boldsymbol{M}, \boldsymbol{\Lambda}, \boldsymbol{\Sigma}, \boldsymbol{\Psi}, W) \leftrightarrow \boldsymbol{\mathcal{Y}}^\top \sim \mathcal{MVMMN}_{n,p}(\boldsymbol{M}^\top, \boldsymbol{\Lambda}^\top, \boldsymbol{\Psi}, \boldsymbol{\Sigma}, W).$$

Proof. Applying transpose property of MVN distribution (theorem 2.3.1 of [22]) into the hierarchical representation (4), the proof is completed.

Theorem 5 *Let* $\boldsymbol{\mathcal{Y}} \sim \mathcal{MVMMN}_{p,n}(\boldsymbol{M}, \boldsymbol{\Lambda}, \boldsymbol{\Sigma}, \boldsymbol{\Psi}, W)$*, and* $\boldsymbol{B}$ *is a* $q \times p$ *matrix of rank* $q \leq p$ *and* $\boldsymbol{D}$ *is a* $n \times m$ *matrix of rank* $m \leq n$*. Then,*

$$\boldsymbol{B}\boldsymbol{\mathcal{Y}}\boldsymbol{D} \sim \mathcal{MVMMN}_{q,m}(\boldsymbol{BMD}, \boldsymbol{B\Lambda D}, \boldsymbol{B\Sigma B}^\top, \boldsymbol{D}^\top\boldsymbol{\Psi D}, W).$$

Proof. The proof of the theorem is completed through obtaining the characteristic function of $\boldsymbol{B\mathcal{Y}D}$.

$$\varphi_{\boldsymbol{B\mathcal{Y}D}}(\boldsymbol{T}) = E\left(\operatorname{etr}\left\{i\boldsymbol{B\mathcal{Y}DT}^\top\right\}\right) = E\left(\operatorname{etr}\left\{i\boldsymbol{\mathcal{Y}T}_1^\top\right\}\right) = \varphi_{\boldsymbol{\mathcal{Y}}}(\boldsymbol{T}_1^\top),$$

where $\boldsymbol{T}_1 = \boldsymbol{DT}^\top\boldsymbol{B}$. Now, by applying Theorem 2, we have

$$\varphi_{\boldsymbol{B\mathcal{Y}D}}(\boldsymbol{T}) = \operatorname{etr}\left\{i\boldsymbol{T}^\top\boldsymbol{B}\mathbf{M}\boldsymbol{D} - \frac{1}{2}\boldsymbol{T}^\top(\boldsymbol{B\Sigma B}^\top)\boldsymbol{T}(\boldsymbol{D}^\top\boldsymbol{\Psi D})\right\}\varphi_W\left(\operatorname{tr}\left(\boldsymbol{T}^\top\boldsymbol{B\Lambda D}\right)\right),$$

which is a characteristic function of $\mathcal{MVMMN}_{q,m}(\boldsymbol{B}\mathbf{M}\boldsymbol{D}, \boldsymbol{B\Lambda D}, \boldsymbol{B\Sigma B}^\top, \boldsymbol{D}^\top\boldsymbol{\Psi D}, W)$.

Theorem 6 *Let* $\boldsymbol{\mathcal{Y}} \sim \mathcal{MVMMN}_{p,n}(\boldsymbol{M}, \boldsymbol{\Lambda}, \boldsymbol{\Sigma}, \boldsymbol{\Psi}, W)$*, and partition* $\boldsymbol{\mathcal{Y}}, \boldsymbol{M}, \boldsymbol{\Lambda}, \boldsymbol{\Sigma}$*, and* $\boldsymbol{\Psi}$ *as*

$$\boldsymbol{\mathcal{Y}} = \begin{pmatrix} \boldsymbol{\mathcal{Y}}_{11} & \boldsymbol{\mathcal{Y}}_{12} \\ \boldsymbol{\mathcal{Y}}_{21} & \boldsymbol{\mathcal{Y}}_{22} \end{pmatrix}, \quad \boldsymbol{M} = \begin{pmatrix} \boldsymbol{M}_{11} & \boldsymbol{M}_{12} \\ \boldsymbol{M}_{21} & \boldsymbol{M}_{22} \end{pmatrix}, \quad \boldsymbol{\Lambda} = \begin{pmatrix} \boldsymbol{\Lambda}_{11} & \boldsymbol{\Lambda}_{12} \\ \boldsymbol{\Lambda}_{21} & \boldsymbol{\Lambda}_{22} \end{pmatrix},$$
$$\boldsymbol{\Sigma} = \begin{pmatrix} \boldsymbol{\Sigma}_{11} & \boldsymbol{\Sigma}_{12} \\ \boldsymbol{\Sigma}_{21} & \boldsymbol{\Sigma}_{22} \end{pmatrix}, \quad \boldsymbol{\Psi} = \begin{pmatrix} \boldsymbol{\Psi}_{11} & \boldsymbol{\Psi}_{12} \\ \boldsymbol{\Psi}_{21} & \boldsymbol{\Psi}_{22} \end{pmatrix},$$

where $\boldsymbol{\mathcal{Y}}_{11}, \boldsymbol{M}_{11}, \boldsymbol{\Lambda}_{11} \in \mathbb{R}^{q\times m}$, $\boldsymbol{\Sigma}_{11} \in \mathbb{R}^{q\times q}$*, and* $\boldsymbol{\Psi}_{11} \in \mathbb{R}^{m\times m}$*. Then,*

$$\boldsymbol{\mathcal{Y}}_{11} \sim \mathcal{MVMMN}_{q,m}(\boldsymbol{M}_{11}, \boldsymbol{\Lambda}_{11}, \boldsymbol{\Sigma}_{11}, \boldsymbol{\Psi}_{11}, W).$$

Similarly, the marginal distribution of $\boldsymbol{\mathcal{Y}}_{12}, \boldsymbol{\mathcal{Y}}_{21}$*, and* $\boldsymbol{\mathcal{Y}}_{22}$ *can be obtained.*

Proof. The proof follows by applying Theorem 5 with considering $\boldsymbol{B} = (\boldsymbol{I}_q \ \ \mathbf{0}_{q\times(q-p)})$ and $\boldsymbol{D} = (\boldsymbol{I}_m \ \ \mathbf{0}_{m\times(n-m)})^\top$, where $\boldsymbol{I}_d$ denotes the unit matrix of order d.

Theorem 7 *Let $\boldsymbol{\mathcal{Y}} \sim \mathcal{MVMMN}_{p,n}(\boldsymbol{M}, \boldsymbol{\Lambda}, \boldsymbol{\Sigma}, \boldsymbol{\Psi}, W)$, and partition $\boldsymbol{\Psi}, \boldsymbol{\Sigma}$ as Theorem 6, and $\boldsymbol{\mathcal{Y}}, \boldsymbol{M}, \boldsymbol{\Lambda}$ as follows*

$$\boldsymbol{\mathcal{Y}} = \begin{pmatrix} \boldsymbol{\mathcal{Y}}_{1r} \\ \boldsymbol{\mathcal{Y}}_{2r} \end{pmatrix} = \left(\boldsymbol{\mathcal{Y}}_{1c}\ \boldsymbol{\mathcal{Y}}_{2c}\right), \quad \boldsymbol{M} = \begin{pmatrix} \boldsymbol{M}_{1r} \\ \boldsymbol{M}_{2r} \end{pmatrix} = \left(\boldsymbol{M}_{1c}\ \boldsymbol{M}_{2c}\right), \quad \boldsymbol{\Lambda} = \begin{pmatrix} \boldsymbol{\Lambda}_{1r} \\ \boldsymbol{\Lambda}_{2r} \end{pmatrix} = \left(\boldsymbol{\Lambda}_{1c}\ \boldsymbol{\Lambda}_{2c}\right),$$

where $\boldsymbol{\mathcal{Y}}_{1r}, \boldsymbol{M}_{1r}, \boldsymbol{\Lambda}_{1r} \in \mathbb{R}^{q\times n}$, and $\boldsymbol{\mathcal{Y}}_{1c}, \boldsymbol{M}_{1c}, \boldsymbol{\Lambda}_{1c} \in \mathbb{R}^{p\times m}$. Then,

(i) $\boldsymbol{\mathcal{Y}}_{1r} \sim \mathcal{MVMMN}_{q,n}\big(\boldsymbol{M}_{1r}, \boldsymbol{\Lambda}_{1r}, \boldsymbol{\Sigma}_{11}, \boldsymbol{\Psi}, W\big)$, and $\boldsymbol{\mathcal{Y}}_{1c} \sim \mathcal{MVMMN}_{p,m}\big(\boldsymbol{M}_{1c}, \boldsymbol{\Lambda}_{1c}, \boldsymbol{\Sigma}, \boldsymbol{\Psi}_{11}, W\big)$.

(ii) $\boldsymbol{\mathcal{Y}}_{2r}|\boldsymbol{\mathcal{Y}}_{1r} = \boldsymbol{Y}_{1r} \sim \mathcal{MVMMN}_{p-q,n}\big(\boldsymbol{M}_{2r}+\boldsymbol{\Sigma}_{21}\boldsymbol{\Sigma}_{11}^{-1}(\boldsymbol{Y}_{1r}-\boldsymbol{M}_{1r}), \boldsymbol{\Lambda}_{2r}-\boldsymbol{\Sigma}_{21}\boldsymbol{\Sigma}_{11}^{-1}\boldsymbol{\Lambda}_{1r}, \boldsymbol{\Sigma}_{22.1}, \boldsymbol{\Psi}, W_{\boldsymbol{Y}_{1r}}\big)$, and $\boldsymbol{\mathcal{Y}}_{2c}|\boldsymbol{\mathcal{Y}}_{1c} = \boldsymbol{Y}_{1c} \sim \mathcal{MVMMN}_{p,n-m}\big(\boldsymbol{M}_{2c} + (\boldsymbol{Y}_{1c} - \boldsymbol{M}_{1c})\boldsymbol{\Psi}_{11}^{-1}\boldsymbol{\Psi}_{12}, \boldsymbol{\Lambda}_{2c} - \boldsymbol{\Lambda}_{1c}\boldsymbol{\Psi}_{11}^{-1}\boldsymbol{\Psi}_{12}, \boldsymbol{\Sigma}, \boldsymbol{\Psi}_{22.1}, W_{\boldsymbol{Y}_{1c}}\big)$, where $\boldsymbol{\Sigma}_{22.1} = \boldsymbol{\Sigma}_{22} - \boldsymbol{\Sigma}_{21}\boldsymbol{\Sigma}_{11}^{-1}\boldsymbol{\Sigma}_{12}$, $\boldsymbol{\Psi}_{22.1} = \boldsymbol{\Psi}_{22} - \boldsymbol{\Psi}_{21}\boldsymbol{\Psi}_{11}^{-1}\boldsymbol{\Psi}_{12}$, $W_{\boldsymbol{Y}_{1r}} \overset{d}{=} W|\boldsymbol{\mathcal{Y}}_{1r} = \boldsymbol{Y}_{1r}$, and $W_{\boldsymbol{Y}_{1c}} \overset{d}{=} W|\boldsymbol{\mathcal{Y}}_{1c} = \boldsymbol{Y}_{1c}$.

Proof. The proof of (i) is completed by considering proper matrix $\boldsymbol{B}$ and $\boldsymbol{D}$ in Theorem 5. Using the hierarchical representation (4) and applying theorem 2.3.12 of [22] the second part of the theorem is proven.

Corollary 2 *If $\boldsymbol{\mathcal{Y}} \sim \mathcal{RMVSN}(\boldsymbol{M}, \boldsymbol{\Lambda}, \boldsymbol{\Sigma}, \boldsymbol{\Psi})$ and under partition of Theorem 7, we have*

(i) $\boldsymbol{\mathcal{Y}}_{2r}|\boldsymbol{\mathcal{Y}}_{1r} = \boldsymbol{Y}_{1r} \sim \mathcal{MVMMN}_{p-q,n}(\boldsymbol{M}_{2r}+\boldsymbol{\Sigma}_{21}\boldsymbol{\Sigma}_{11}^{-1}(\boldsymbol{Y}_{1r}-\boldsymbol{M}_{1r}), \boldsymbol{\Lambda}_{2r}-\boldsymbol{\Sigma}_{21}\boldsymbol{\Sigma}_{11}^{-1}\boldsymbol{\Lambda}_{1r}, \boldsymbol{\Sigma}_{22.1}, \boldsymbol{\Psi}, W_{\boldsymbol{Y}_{1r}})$ where $W_{\boldsymbol{Y}_{1r}} \sim \mathcal{TN}\left(\boldsymbol{A}_{1r}/\boldsymbol{\eta}_{1r}, 1/\boldsymbol{\eta}_{1r}^2; (0,\infty)\right)$, $\boldsymbol{\eta}_{1r} = tr\left(\boldsymbol{\Psi}^{-1}\boldsymbol{\Lambda}_{1r}^\top\boldsymbol{\Sigma}_{1r}^{-1}\boldsymbol{\Lambda}_{1r}\right)+1$ and $\boldsymbol{A}_{1r} = \left[tr\left(\boldsymbol{\Psi}^{-1}\boldsymbol{\Lambda}_{1r}^\top\boldsymbol{\Sigma}_{1r}^{-1}(\boldsymbol{Y}_{1r} - \boldsymbol{M}_{1r})\right)\right]/\boldsymbol{\eta}_{1r}$.

(ii) $\boldsymbol{\mathcal{Y}}_{2c}|\boldsymbol{\mathcal{Y}}_{1c} = \boldsymbol{Y}_{1c} \sim \mathcal{MVMMN}_{p,n-m}(\boldsymbol{M}_{2c}+(\boldsymbol{Y}_{1c}-\boldsymbol{M}_{1c})\boldsymbol{\Psi}_{11}^{-1}\boldsymbol{\Psi}_{12}, \boldsymbol{\Lambda}_{2c}-\boldsymbol{\Lambda}_{1c}\boldsymbol{\Psi}_{11}^{-1}\boldsymbol{\Psi}_{12}, \boldsymbol{\Sigma}, \boldsymbol{\Psi}_{22.1}, W_{\boldsymbol{Y}_{1c}})$, where $W_{\boldsymbol{Y}_{1c}} \sim \mathcal{TN}\left(\boldsymbol{A}_{1c}/\boldsymbol{\eta}_{1c}, 1/\boldsymbol{\eta}_{1c}^2; (0,\infty)\right)$, $\boldsymbol{\eta}_{1c} = tr\left(\boldsymbol{\Psi}_{1c}^{-1}\boldsymbol{\Lambda}_{1c}^\top\boldsymbol{\Sigma}^{-1}\boldsymbol{\Lambda}_{1c}\right)+1$, and $\boldsymbol{A}_{1c} = \left[tr\left(\boldsymbol{\Psi}_{1c}^{-1}\boldsymbol{\Lambda}_{1c}^\top\boldsymbol{\Sigma}^{-1}(\boldsymbol{Y}_{1c} - \boldsymbol{M}_{1c})\right)\right]/\boldsymbol{\eta}_{1c}$.

Corollary 3 *If $\boldsymbol{\mathcal{Y}} \sim \mathcal{MVMMNE}(\boldsymbol{M}, \boldsymbol{\Lambda}, \boldsymbol{\Sigma}, \boldsymbol{\Psi})$ and under partition of Theorem (7), we have*

(i) $\boldsymbol{\mathcal{Y}}_{2r}|\boldsymbol{\mathcal{Y}}_{1r} = \boldsymbol{Y}_{1r} \sim \mathcal{MVMMN}_{p-q,n}(\boldsymbol{M}_{2r}+\boldsymbol{\Sigma}_{21}\boldsymbol{\Sigma}_{11}^{-1}(\boldsymbol{Y}_{1r}-\boldsymbol{M}_{1r}), \boldsymbol{\Lambda}_{2r}-\boldsymbol{\Sigma}_{21}\boldsymbol{\Sigma}_{11}^{-1}\boldsymbol{\Lambda}_{1r}, \boldsymbol{\Sigma}_{22.1}, \boldsymbol{\Psi}, W_{\boldsymbol{Y}_{1r}})$ where $W_{\boldsymbol{Y}_{1r}} \sim \mathcal{TN}\left(\boldsymbol{A}_{1r}^*/\boldsymbol{\eta}_{1r}^*, 1/\boldsymbol{\eta}_{1r}^{*2}; (0,\infty)\right)$, $\boldsymbol{A}_{1r}^* = \left[tr\left(\boldsymbol{\Psi}^{-1}\boldsymbol{\Lambda}_{1r}^\top\boldsymbol{\Sigma}_{1r}^{-1}(\boldsymbol{Y}_{1r} - \boldsymbol{M}_{1r})\right) - 1\right]/\boldsymbol{\eta}_{1r}^*$, and $\boldsymbol{\eta}_{1r}^* = tr\left(\boldsymbol{\Psi}^{-1}\boldsymbol{\Lambda}_{1r}^\top\boldsymbol{\Sigma}_{1r}^{-1}\boldsymbol{\Lambda}_{1r}\right)$.

(ii) $\boldsymbol{\mathcal{Y}}_{2c}|\boldsymbol{\mathcal{Y}}_{1c} = \boldsymbol{Y}_{1c} \sim \mathcal{MVMMN}_{p,n-m}(\boldsymbol{M}_{2c}+(\boldsymbol{Y}_{1c}-\boldsymbol{M}_{1c})\boldsymbol{\Psi}_{11}^{-1}\boldsymbol{\Psi}_{12}, \boldsymbol{\Lambda}_{2c}-\boldsymbol{\Lambda}_{1c}\boldsymbol{\Psi}_{11}^{-1}\boldsymbol{\Psi}_{12}, \boldsymbol{\Sigma}, \boldsymbol{\Psi}_{22.1}, W_{\boldsymbol{Y}_{1c}})$, where $W_{\boldsymbol{Y}_{1c}} \sim \mathcal{TN}\left(\boldsymbol{A}_{1c}^*/\boldsymbol{\eta}_{1c}^*, 1/\boldsymbol{\eta}_{1c}^{*2}; (0,\infty)\right)$, $\boldsymbol{A}_{1c}^* = \left[tr\left(\boldsymbol{\Psi}_{1c}^{-1}\boldsymbol{\Lambda}_{1c}^\top\boldsymbol{\Sigma}^{-1}(\boldsymbol{Y}_{1c} - \boldsymbol{M}_{1c})\right) - 1\right]/\boldsymbol{\eta}_{1c}^*$, and $\boldsymbol{\eta}_{1c}^* = tr\left(\boldsymbol{\Psi}_{1c}^{-1}\boldsymbol{\Lambda}_{1c}^\top\boldsymbol{\Sigma}^{-1}\boldsymbol{\Lambda}_{1c}\right)$.

The Work of [23] on the distribution of matrix quadratic form can be extended to the family of MVMMN distributions. Referring to theorem 2.2 of [23], they defined the distribution of quadratic form $\mathcal{Q} = \boldsymbol{\mathcal{X}}\mathbf{A}\boldsymbol{\mathcal{X}}^\top$ to be $\mathcal{Q}_p(\mathbf{A}, \mathbf{M}, \boldsymbol{\Sigma})$ where $\mathbf{A}$ is a $n\times n$ symmetric real matrix of rank r, and $\boldsymbol{\mathcal{X}} \sim \mathcal{N}_{p,n}(\mathbf{M}, \boldsymbol{\Sigma}, \boldsymbol{I}_n)$.

Theorem 8 *Let $\boldsymbol{\mathcal{Y}} \sim \mathcal{MVMMN}_{p,n}(\boldsymbol{M}, \boldsymbol{\Lambda}, \boldsymbol{\Sigma}, \boldsymbol{\Psi}, W)$ and $W \sim h(w; \boldsymbol{\nu})$ and $\boldsymbol{A}_{n\times n}$ any $n \times n$ symmetric matrix of rank r. Then, conditionally on $W = w$,*

$$\mathcal{Q} = \boldsymbol{\mathcal{Y}}\boldsymbol{A}\boldsymbol{\mathcal{Y}}^\top|W = w \qquad \textit{and} \qquad \boldsymbol{B} = \sum_{j=1}^{r}\delta_j\boldsymbol{B}_j|W = w,$$

are identically distributed, where δ_j are the non-zero eigenvalues of $\boldsymbol{\Psi}^{-\frac{1}{2}}\boldsymbol{A}\boldsymbol{\Psi}^{-\frac{1}{2}}$ and $\boldsymbol{B}_j$ are independent non-central Wishart distribution $\boldsymbol{B}_j|W = w \sim \mathcal{W}_p(1, \boldsymbol{\Sigma}, \boldsymbol{m}_j\boldsymbol{m}_j^\top)$ for $j = 1, \ldots, r$, where $\boldsymbol{m}_j = \boldsymbol{M}\boldsymbol{a}_j$ and $\boldsymbol{a}_j$ are the corresponding orthogonal eigenvectors ($\boldsymbol{a}_j^\top\boldsymbol{a}_j = 1$).

Proof. The Theorem can be pr[illegible] by using hierarchical representation (4) and applying theorem 2.2 of [23]. Note furthermore that the distribution of $\boldsymbol{\mathcal{Y}}\boldsymbol{\Psi}^{-\frac{1}{2}}\mathbf{A}\boldsymbol{\Psi}^{-\frac{1}{2}}\boldsymbol{\mathcal{Y}}$ conditionally on $W = w$, is $\mathcal{Q}_p(\mathbf{A}, \mathbf{M}\boldsymbol{\Psi}^{-\frac{1}{2}} + w\boldsymbol{\Lambda}\boldsymbol{\Psi}^{-\frac{1}{2}}, \boldsymbol{\Sigma})$. If $\mathbf{A}$ is idempotent then $\mathcal{Q}_p$ simplifies the non-central Wishart distribution.

4 Parameter estimation

Suppose N matrix observations $\mathbf{Y}_1, \ldots, \mathbf{Y}_N$ of dimension $p \times n$ are drawn independently and identically from the $\mathcal{MVMMN}_{p,n}(\mathbf{M}, \boldsymbol{\Lambda}, \boldsymbol{\Sigma}, \boldsymbol{\Psi}, W)$. Therefore, the log-likelihood function of $\boldsymbol{\Theta} = (\mathbf{M}, \boldsymbol{\Lambda}, \boldsymbol{\Sigma}, \boldsymbol{\Psi}, \boldsymbol{\nu})$ based on the observed data $\{\mathbf{Y}_i\}_{i=1}^N$ is

$$\ell(\boldsymbol{\Theta}) = \sum_{i=1}^{N} \log f(\mathbf{Y}_i; \mathbf{M}, \boldsymbol{\Lambda}, \boldsymbol{\Sigma}, \boldsymbol{\Psi}, \boldsymbol{\nu}). \tag{7}$$

To obtain ML estimate of $\boldsymbol{\Theta}$, the [illegible]-type algorithm is implemented as a powerful estimation approach in dealing with the unobserved (missing and/or censored) data and latent variables. The computations of EM algorithm are based on two iterative E- and M-steps. In the E-step, the expected value of the complete-data log-likelihood function, the likelihood of the observed and missing data the latent variable, is computed, while in the M-step, parameter estimates are updated by maximizing this expected value.

Through the hierarchical representation (4), the complete-data log-likelihood function of $\boldsymbol{\Theta}$, obtained by introducing latent variables $\boldsymbol{W} = (w_1, \ldots, w_N)$ and omitting additive constants, is

$$\begin{aligned}\ell_c(\boldsymbol{\Theta}) = &\sum_{i=1}^{N} \log h(w_i; \boldsymbol{\nu}) - \frac{nN}{2} \log|\boldsymbol{\Sigma}| - \frac{pN}{2} \log|\boldsymbol{\Psi}| \\ &- \frac{1}{2}\sum_{i=1}^{N} \Bigg\{ \mathrm{tr}\left(\boldsymbol{\delta}(\mathbf{Y}_i, \mathbf{M}, \boldsymbol{\Psi}, \boldsymbol{\Sigma})\right) + w_i^2 \mathrm{tr}\left(\boldsymbol{\Psi}^{-1}\boldsymbol{\Lambda}^\top\boldsymbol{\Sigma}^{-1}\boldsymbol{\Lambda}\right) \\ &\qquad - w_i\Big(\mathrm{tr}\left(\boldsymbol{\Psi}^{-1}\boldsymbol{\Lambda}^\top\boldsymbol{\Sigma}^{-1}\big(\mathbf{Y}_i - \mathbf{M}\big)\right) + \mathrm{tr}\left(\boldsymbol{\Psi}^{-1}\big(\mathbf{Y}_i - \mathbf{M}\big)^\top\boldsymbol{\Sigma}^{-1}\boldsymbol{\Lambda}\right)\Big)\Bigg\}. \end{aligned} \tag{8}$$

ML estimation of $\boldsymbol{\Theta}$ is performed by using the expectation-conditional maximization (ECM; [24]) algorithm as follows.

- Initialization: Set the number of iteration to $k = 0$ and choose a relative starting point $\boldsymbol{\Theta}^{(k)} = (\mathbf{M}^{(k)}, \boldsymbol{\Lambda}^{(k)}, \boldsymbol{\Sigma}^{(k)}, \boldsymbol{\Psi}^{(k)}, \boldsymbol{\nu}^{(k)})$. We point out that in our data examples the parameters are initialized by $\mathbf{M}^{(0)} = \sum_{i=1}^{N} \mathbf{Y}_i/N$, $\boldsymbol{\Lambda}^{(0)} = \mathbf{1}_{p\times n}$, $\boldsymbol{\Sigma}^{(0)} = c_1\boldsymbol{I}_p$, $\boldsymbol{\Psi}^{(0)} = c_2\boldsymbol{I}_n$. Here, $\mathbf{1}_{p\times n}$ is a matrix of dimension $p \times n$ with unit elements. Moreover, the elements of two vectors c_1 and c_2 are computed, respectively, as

$$c_{1_j} = \sum_{i=1}^{N}\sum_{l=1}^{n}(y_{ijl} - \bar{y}_j)^2, \qquad \bar{y}_j = \frac{1}{nN}\sum_{i=1}^{N}\sum_{l=1}^{n} y_{ijl}, \qquad j = 1, \ldots, p,$$
$$c_{2_l} = \sum_{i=1}^{N}\sum_{j=1}^{p}(y_{ijl} - \bar{y}_l)^2, \qquad \bar{y}_l = \frac{1}{pN}\sum_{i=1}^{N}\sum_{j=1}^{p} y_{ijl}, \qquad j = 1, \ldots, n.$$

- E-step: The expected value of the complete-data log-likelihood function (8), called

Q-function, is computed as

$$
\begin{aligned}
Q(\boldsymbol{\Theta}|\hat{\boldsymbol{\Theta}}^{(k)}) = & \sum_{i=1}^{N} \hat{\Upsilon}_i^{(k)} - \frac{nN}{2}\log|\boldsymbol{\Sigma}| - \frac{pN}{2}\log|\boldsymbol{\Psi}| \\
& - \frac{1}{2}\sum_{i=1}^{N}\Bigg\{ \mathrm{tr}\left(\boldsymbol{\delta}(\mathbf{Y}_i, \mathbf{M}, \boldsymbol{\Psi}, \boldsymbol{\Sigma})\right) + \hat{t}_i^{(k)}\mathrm{tr}\left(\boldsymbol{\Psi}^{-1}\boldsymbol{\Lambda}^{\top}\boldsymbol{\Sigma}^{-1}\boldsymbol{\Lambda}\right) \\
& - \hat{w}_i^{(k)}\Big(\mathrm{tr}\left(\boldsymbol{\Psi}^{-1}\boldsymbol{\Lambda}^{\top}\boldsymbol{\Sigma}^{-1}\big(\mathbf{Y}_i - \mathbf{M}\big)\right) + \mathrm{tr}\left(\boldsymbol{\Psi}^{-1}\big(\mathbf{Y}_i - \mathbf{M}\big)^{\top}\boldsymbol{\Sigma}^{-1}\boldsymbol{\Lambda}\right)\Big)\Bigg\}, \quad (9)
\end{aligned}
$$

where $\hat{w}_i^{(k)} = E(W|\mathbf{Y}_i, \hat{\boldsymbol{\Theta}}^{(k)})$, $\hat{t}_i^{(k)} = E(W^2|\mathbf{Y}_i, \hat{\boldsymbol{\Theta}}^{(k)})$, and depending on $h(w;\boldsymbol{\nu})$ $\hat{\Upsilon}_i^{(k)} = E(\log h(W;\boldsymbol{\nu})|\mathbf{Y}_i, \hat{\boldsymbol{\Theta}}^{(k)})$.

- First CM-step: Maximizing Q-function with respect to $\mathbf{M}$ and $\boldsymbol{\Lambda}$ give the following update

$$
\hat{\mathbf{M}}^{(k+1)} = \frac{1}{N}\sum_{i=1}^{N}\mathbf{Y}_i - \hat{\boldsymbol{\Lambda}}^{(k)}\bar{w}, \qquad \hat{\boldsymbol{\Lambda}}^{(k+1)} = \frac{N^{-1}\sum_{i=1}^{N}\hat{w}_i^{(k)}\mathbf{Y}_i - N^{-1}\bar{w}\sum_{i=1}^{N}\mathbf{Y}_i}{\bar{t} - \bar{w}^2},
$$

where $\bar{w} = N^{-1}\sum_{i=1}^{N}\hat{w}_i^{(k)}$ and $\bar{t} = N^{-1}\sum_{i=1}^{N}\hat{t}_i^{(k)}$.

- Second CM-step: Update $\boldsymbol{\Sigma}$ and $\boldsymbol{\Psi}$, respectively,

$$
\begin{aligned}
\hat{\boldsymbol{\Sigma}}^{(k+1)} = & \frac{1}{Nn}\sum_{i=1}^{N}\Bigg\{\left(\mathbf{Y}_i - \hat{\mathbf{M}}^{(k+1)}\right)\left(\hat{\boldsymbol{\Psi}}^{(k)}\right)^{-1}\left(\mathbf{Y}_i - \hat{\mathbf{M}}^{(k+1)}\right)^{\top} \\
& + \hat{t}_i^{(k)}\hat{\boldsymbol{\Lambda}}^{(k+1)}\big(\hat{\boldsymbol{\Psi}}^{(k)}\big)^{-1}\big(\hat{\boldsymbol{\Lambda}}^{(k+1)}\big)^{\top} - \hat{w}_i^{(k)}\hat{\boldsymbol{\Lambda}}^{(k+1)}\big(\hat{\boldsymbol{\Psi}}^{(k)}\big)^{-1}\left(\mathbf{Y}_i - \hat{\mathbf{M}}^{(k+1)}\right)^{\top} \\
& - \hat{w}_i^{(k)}\left(\mathbf{Y}_i - \hat{\mathbf{M}}^{(k+1)}\right)\big(\hat{\boldsymbol{\Psi}}^{(k)}\big)^{-1}\big(\hat{\boldsymbol{\Lambda}}^{(k+1)}\big)^{\top}\Bigg\}, \\
\hat{\boldsymbol{\Psi}}^{(k+1)} = & \frac{1}{Np}\sum_{i=1}^{N}\Bigg\{\left(\mathbf{Y}_i - \hat{\mathbf{M}}^{(k+1)}\right)^{\top}\big(\hat{\boldsymbol{\Sigma}}^{(k+1)}\big)^{-1}\left(\mathbf{Y}_i - \hat{\mathbf{M}}^{(k+1)}\right) \\
& + \hat{t}_i^{(k)}\big(\hat{\boldsymbol{\Lambda}}^{(k+1)}\big)^{\top}\big(\hat{\boldsymbol{\Sigma}}^{(k+1)}\big)^{-1}\hat{\boldsymbol{\Lambda}}^{(k+1)} \\
& - \hat{w}_i^{(k)}\big(\hat{\boldsymbol{\Lambda}}^{(k+1)}\big)^{\top}\big(\hat{\boldsymbol{\Sigma}}^{(k+1)}\big)^{-1}\left(\mathbf{Y}_i - \hat{\mathbf{M}}^{(k+1)}\right) \\
& - \hat{w}_i^{(k)}\left(\mathbf{Y}_i - \hat{\mathbf{M}}^{(k+1)}\right)^{\top}\big(\hat{\boldsymbol{\Sigma}}^{(k+1)}\big)^{-1}\hat{\boldsymbol{\Lambda}}^{(k+1)}\Bigg\}.
\end{aligned}
$$

- Third CM-step: The additional parameter $\boldsymbol{\nu}$ depending on the distribution of W_i is updated by

$$
\hat{\boldsymbol{\nu}}^{(k+1)} = \arg\max_{\boldsymbol{\nu}}\sum_{i=1}^{N}\hat{\Upsilon}_i^{(k)}.
$$

Remark 2 *The conditional expectations $\hat{w}_i$ and $\hat{t}_i$ involved in the Q-function (9) can be obtained by Lemma 1 and Propositions 1, 2 and 3 for our three considered models. Furthermore, we note that in all special cases considered in Section 4, the distribution of mixing random variable W, is parameter free. Therefore, the last step of the ECM algorithm is not necessary.*

4.1 Computational aspects

4.1.1 Convergence

The process of the EM algorithm can be iterated until a suitable convergence rule, like $\max \|\hat{\mathbf{\Theta}}^{(k+1)} - \hat{\mathbf{\Theta}}^{(k)}\| \leq \varepsilon$ or $|\ell(\hat{\mathbf{\Theta}}^{(k+1)}) - \ell(\hat{\mathbf{\Theta}}^{(k)})| \leq \varepsilon$, is satisfied where ε is a user specified tolerance and $\ell(\cdot)$ is the log-likelihood function defined in (7). An alternative approach to determine convergence of the EM algorithm is the Aitken acceleration [25]. To apply this approach, the asymptotic estimate of the log-likelihood at iteration $k+1$, following [26], can be obtained as

$$\ell_\infty(\hat{\mathbf{\Theta}}^{(k+1)}) = \ell(\hat{\mathbf{\Theta}}^{(k+1)}) + \frac{1}{1-a^{(k)}}\Big(\ell(\hat{\mathbf{\Theta}}^{(k+1)}) - \ell(\hat{\mathbf{\Theta}}^{(k)})\Big),$$

where the Aitken acceleration of iteration k is

$$a^{(k)} = \frac{\ell(\hat{\mathbf{\Theta}}^{(k+1)}) - \ell(\hat{\mathbf{\Theta}}^{(k)})}{\ell(\hat{\mathbf{\Theta}}^{(k)}) - \ell(\hat{\mathbf{\Theta}}^{(k-1)})}.$$

Therefore, the algorithm can be considered to have converged at iteration $k+1$ when $\ell_\infty(\hat{\mathbf{\Theta}}^{(k+1)}) - \ell(\hat{\mathbf{\Theta}}^{(k)}) < \epsilon$, [27]. In our study, the tolerance ϵ is considered as 10^{-5}.

4.1.2 Model Selection

The models in competition in our data analysis are compared using the most commonly used measure Akaike information criterion (AIC; [28]) and Bayesian information criterion (BIC; [29]) defined as

$$\text{AIC} = 2m - 2\ell_{\max} \quad \text{and} \quad \text{BIC} = m\log N - 2\ell_{\max},$$

where m is the number of free parameters and $\ell_{\max}$ is the maximized log-likelihood value. Models with lower values of AIC or BIC are considered more preferable.

5 Simulation studies

In this section, the performance of our model and its computational method is illustrated by conducting tw[illegible]mulation studies. The first simulation study aims at comparing the special cases of MVMMN model in dealing with skewed and leptokurtic simulated data. The second simulation study demonstrates whether our proposed ECM algorithm can provide good asymptotic properties.

Example 1 Model performance

In this experiment, artificial data are generated from a matrix variate normal inverse Gaussian (MVNIG; [20]) distribution with various sample si[illegible] $n = 50, 100, 500, 1000$ *and 2000, to compare the performance of three special cases* [illegible]*MVMMN model. The MVNIG distribution belongs to the family of MVMVM models where the mixing random variable follows the* $\mathcal{GIG}(-0.5, \chi, \psi)$*, such that* $\mathcal{GIG}$ *denotes the generalized inverse Gaussian distribution with parameter* (κ, χ, ψ) *[30]. We consider this matrix variate distribution to generate non-normal data as it offers the desired level of asymmetry and leptokurtosis. Let* $\chi = \psi = 3$ *and*

$$\boldsymbol{M} = \begin{bmatrix} -5 & 2 & 0 & 2 \\ -2 & 0 & 3 & 0 \\ 0 & 1 & 6 & -4 \end{bmatrix}, \quad \mathbf{\Lambda} = \begin{bmatrix} 1 & -1 & 0 & 1 \\ 2 & -1 & 0 & -2 \\ 0 & -1 & 0 & -3 \end{bmatrix},$$

$$\mathbf{\Sigma} = \begin{bmatrix} 1 & -0.5 & 0.1 \\ -0.5 & 1 & 0.5 \\ 0.1 & 0.5 & 1 \end{bmatrix}, \quad \mathbf{\Psi} = \begin{bmatrix} 1 & 0 & 0 & 0 \\ 0 & 1 & -0.5 & 0.5 \\ 0 & -0.5 & 1 & 0.1 \\ 0 & 0.5 & 0.1 & 1 \end{bmatrix}.$$

Table 1. Mean of maximized log-likelihood with its standard deviation and frequency of model outperformance in 200 replications with various sample sizes.

	RMVSN			MVMMNE			MVMMNW		
N	ℓ_{max}	Sd	Freq.	ℓ_{max}	Sd	Freq.	ℓ_{max}	Sd	Freq.
50	-731.14	35.60	55	-729.90	35.54	141	-733.99	35.82	4
100	-1503.40	43.96	46	-1501.06	43.65	154	-1508.61	43.98	0
500	-7622.68	114.60	14	-7610.95	113.25	186	-7649.34	115.98	0
1000	-15278.40	147.93	2	-15254.32	145.88	197	-15329.81	148.41	1
2000	-30574.13	206.84	1	-30528.32	205.33	198	-30680.56	206.61	1

Table 2. Frobenius norm for parameter estimates of the RMVSN, MVMMNE and MVMMNW distributions as a function of sample size.

parameter→	$\boldsymbol{M}$			$\boldsymbol{\Lambda}$		
$N\downarrow$	RMVSN	MVMMNE	MVMMNW	RMVSN	MVMMNE	MVMMNW
50	1.7135	2.0228	1.1425	1.3717	2.1097	1.5439
100	1.6714	2.0197	0.8540	1.0385	2.0324	1.0986
500	1.5130	1.8976	0.3955	0.6317	1.9081	0.6722
1000	1.4822	1.8772	0.3033	0.5559	1.8806	0.6163
2000	1.4796	1.864 1	0.2460	0.5268	1.8752	0.5931
parameter→	$\boldsymbol{\Sigma}$			$\boldsymbol{\Psi}$		
$N\downarrow$	RMVSN	MVMMNE	MVMMNW	RMVSN	MVMMNE	MVMMNW
50	0.3536	0.3546	0.3520	0.4378	0.4370	0.4361
100	0.2311	0.2314	0.2300	0.2997	0.3000	0.2986
500	0.1043	0.1039	0.1044	0.1300	0.1291	0.1303
1000	0.0744	0.0739	0.0741	0.0981	0.0976	0.0979
2000	0.0515	0.0511	0.0513	0.0707	0.0701	0.0702

Table 1 summarizes the mean and standard deviation (Sd) of the maximized log-likelihood together with the frequencies (out of 200 replications) of the particular model chosen based on the biggest ℓ_{max} value. The results depicted in Table 1 reveal that the MVMMNE distribution provides a better fit than the other two MVMMN-based models. It is clear that the outperformance of MVMMNE distribution is improved by increasing the sample size.

In order to compare the accuracy of parameter estimates to the real values, we compute the Frobenius (Frob.) norm in each replication. Table 2 shows the average Frob. norm of $\boldsymbol{M}, \boldsymbol{\Lambda}, \boldsymbol{\Sigma}$ and $\boldsymbol{\Psi}$ for all three considered models as a function of sample size. It is observe that the Frob. norm decreases when the sample size increases. We can also see that the Frob. norm for $\boldsymbol{\Sigma}$ and $\boldsymbol{\Psi}$ for all models are very close to each other while the MVMME model has the furthest estimates of $\boldsymbol{M}$ and $\boldsymbol{\Lambda}$.

Example 2 Finite sample properties of the ML estimates

The second simulation study aims at investigating the finite-sample properties of ML estimators obtained by using the ECM algorithm. We consider the situation where Monte Carlo samples of sizes $n = 100$ and 500 are generated for each of the three special cases of MVMMN distribution. The presumed parameters for all distributions are same as used in Example 1. Figure 1 shows the marginal distributions of the columns, labeled by $V1, V2, V3$, and $V4$, for the RMVSN, MVMMNE and MVMMNW distributions of a typical dataset with size 100. The solid red line highlights the marginal mean. In each replication of 1000 trials, the synthetic dataset was fitted with the true generator model via ECM algorithm. To investigate the estimation accuracies, we calculate the bias and the mean squared error (MSE), defined as

$$Bias = \frac{1}{1000}\sum_{k=1}^{1000}\hat{\theta}_k - \theta_{true} \quad and \quad MSE = \frac{1}{1000}\sum_{k=1}^{1000}\left(\hat{\theta}_k - \theta_{true}\right)^2,$$

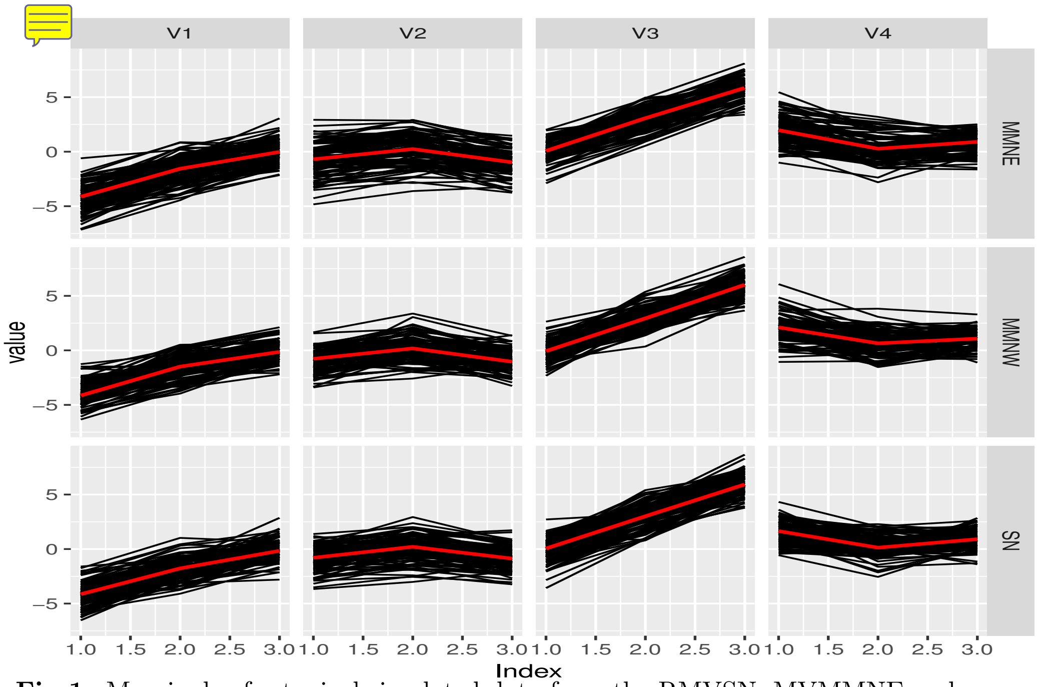

Fig 1. Marginals of a typical simulated data form the RMVSN, MVMMNE and MVMMNW distributions.

where $\hat{\theta}_k$ denotes the ML estimate of θ_{true} (a specific parameter) at the kth replication.

The detailed numerical results are reported in Table 3. It can be observed that the bias and MSE for all three special cases of MVMMN distribution tend to decrease toward zero by increasing the sample size, showing empirically the consistency of the ML estimates obtained via the ECM algorithm.

6 Analysis of Landsat data

To investigate the performance of the developed model in real-world data analysis, we consider landsat satellite data (LSD) originally obtained by NASA and available at Irvine machine learning repository (http://archive.ics.uci.edu/ml). Each line of the LSD contains of four spectral values of nine pixel neighborhoods in a satellite image. In other words, the lines of LSD are related to a matrix of observations of 4×9 dimension. Moreover, each of the LSD matrix of observations belongs to one of six different classes, namely red soil, cotton crop, grey soil, damp grey soil, soil with vegetation stubble, and very damp grey soil. In our analysis, we focus on two classes, red soil and cotton crop, with size 461 and 224, respectively.

We fitted RMMVSN, MVMMNE and MVMMNW distributions by implementing the ECM algorithm. Table 4 shows a summary of ML fitting results, including the parameter estimates, maximized log-likelihood values, AIC and BIC of the three fitted models. It is observed that the MVMMNW and MVMMNE distributions respectively outperform the others for the red soil and cotton crop data. Based on the values of the shape matrix $\mathbf{\Lambda}$, it is clear that the estimated skewness parameters are moderately to highly significant, showing that the distribution of matrix observation is skewed. Moreover, the estimated scale matrices $\mathbf{\Sigma}$ and $\mathbf{\Psi}$ highlight the covariance structure in the data.

Table 3. Simulation results for assessing the consistency of ML parameter estimates with two sample sizes.

Model	N	Measure	$\mathbf{M}$	$\boldsymbol{\Lambda}$	$\boldsymbol{\Sigma}$	$\boldsymbol{\Psi}$
RMVSN	100	Bias	$\begin{bmatrix} 0.015 & 0.003 & -0.002 & 0.015 \\ -0.008 & -0.014 & 0.005 & -0.018 \\ -0.007 & -0.007 & 0.002 & -0.010 \end{bmatrix}$	$\begin{bmatrix} -0.019 & -0.001 & 0.001 & -0.015 \\ 0.012 & 0.014 & -0.009 & 0.015 \\ 0.009 & 0.006 & -0.007 & 0.008 \end{bmatrix}$	$\begin{bmatrix} -0.023 & 0.010 & -0.003 \\ 0.010 & -0.024 & -0.014 \\ -0.003 & -0.014 & -0.025 \end{bmatrix}$	$\begin{bmatrix} -0.001 & -0.002 & 0.004 & -0.002 \\ -0.002 & 0.007 & -0.003 & 0.004 \\ 0.004 & -0.003 & 0.003 & -0.002 \\ -0.002 & 0.004 & -0.002 & 0.003 \end{bmatrix}$
		MSE	$\begin{bmatrix} 0.030 & 0.030 & 0.030 & 0.028 \\ 0.035 & 0.030 & 0.031 & 0.037 \\ 0.030 & 0.029 & 0.030 & 0.042 \end{bmatrix}$	$\begin{bmatrix} 0.036 & 0.036 & 0.030 & 0.034 \\ 0.055 & 0.037 & 0.031 & 0.055 \\ 0.029 & 0.035 & 0.031 & 0.083 \end{bmatrix}$	$\begin{bmatrix} 0.006 & 0.003 & 0.003 \\ 0.003 & 0.006 & 0.004 \\ 0.003 & 0.004 & 0.007 \end{bmatrix}$	$\begin{bmatrix} 0.007 & 0.004 & 0.004 & 0.005 \\ 0.004 & 0.006 & 0.004 & 0.005 \\ 0.004 & 0.004 & 0.005 & 0.004 \\ 0.005 & 0.005 & 0.004 & 0.007 \end{bmatrix}$
	500	Bias	$\begin{bmatrix} 0.004 & 0.001 & -0.001 & 0.001 \\ -0.002 & 0.001 & -0.001 & -0.003 \\ 0.000 & 0.004 & 0.000 & -0.001 \end{bmatrix}$	$\begin{bmatrix} -0.005 & 0.000 & 0.000 & 0.000 \\ 0.004 & 0.000 & 0.000 & 0.001 \\ 0.000 & -0.004 & 0.003 & -0.001 \end{bmatrix}$	$\begin{bmatrix} -0.004 & 0.002 & 0.001 \\ 0.002 & -0.004 & -0.002 \\ 0.001 & -0.002 & -0.003 \end{bmatrix}$	$\begin{bmatrix} -0.001 & -0.001 & 0.001 & 0.000 \\ -0.001 & 0.002 & -0.001 & 0.003 \\ 0.001 & -0.001 & 0.000 & -0.001 \\ 0.000 & 0.003 & -0.001 & 0.002 \end{bmatrix}$
		MSE	$\begin{bmatrix} 0.006 & 0.006 & 0.006 & 0.006 \\ 0.007 & 0.006 & 0.006 & 0.007 \\ 0.006 & 0.006 & 0.006 & 0.009 \end{bmatrix}$	$\begin{bmatrix} 0.007 & 0.007 & 0.006 & 0.007 \\ 0.011 & 0.007 & 0.006 & 0.010 \\ 0.006 & 0.007 & 0.006 & 0.016 \end{bmatrix}$	$\begin{bmatrix} 0.001 & 0.001 & 0.001 \\ 0.001 & 0.001 & 0.001 \\ 0.001 & 0.001 & 0.001 \end{bmatrix}$	$\begin{bmatrix} 0.001 & 0.001 & 0.001 & 0.001 \\ 0.001 & 0.001 & 0.001 & 0.001 \\ 0.001 & 0.001 & 0.001 & 0.001 \\ 0.001 & 0.001 & 0.001 & 0.001 \end{bmatrix}$
MVMMNE	100	Bias	$\begin{bmatrix} 0.015 & -0.010 & -0.004 & 0.004 \\ 0.011 & 0.000 & 0.003 & -0.009 \\ 0.005 & 0.003 & -0.002 & -0.016 \end{bmatrix}$	$\begin{bmatrix} -0.022 & 0.021 & 0.002 & -0.021 \\ -0.039 & 0.018 & -0.003 & 0.036 \\ 0.002 & 0.016 & -0.003 & 0.057 \end{bmatrix}$	$\begin{bmatrix} -0.017 & 0.009 & 0.004 \\ 0.009 & -0.017 & -0.011 \\ 0.004 & -0.011 & -0.018 \end{bmatrix}$	$\begin{bmatrix} 0.001 & -0.003 & 0.005 & 0.000 \\ -0.003 & 0.008 & -0.006 & 0.003 \\ 0.005 & -0.006 & 0.005 & -0.003 \\ 0.000 & 0.003 & -0.003 & -0.001 \end{bmatrix}$
		MSE	$\begin{bmatrix} 0.021 & 0.020 & 0.020 & 0.019 \\ 0.023 & 0.020 & 0.019 & 0.024 \\ 0.020 & 0.018 & 0.018 & 0.030 \end{bmatrix}$	$\begin{bmatrix} 0.020 & 0.020 & 0.011 & 0.018 \\ 0.047 & 0.019 & 0.011 & 0.045 \\ 0.011 & 0.018 & 0.011 & 0.092 \end{bmatrix}$	$\begin{bmatrix} 0.005 & 0.003 & 0.003 \\ 0.003 & 0.006 & 0.004 \\ 0.003 & 0.004 & 0.006 \end{bmatrix}$	$\begin{bmatrix} 0.006 & 0.004 & 0.004 & 0.004 \\ 0.004 & 0.006 & 0.005 & 0.004 \\ 0.004 & 0.005 & 0.006 & 0.003 \\ 0.004 & 0.004 & 0.003 & 0.006 \end{bmatrix}$
	500	Bias	$\begin{bmatrix} 0.000 & 0.000 & -0.003 & 0.001 \\ 0.003 & 0.000 & 0.002 & 0.002 \\ 0.001 & 0.002 & -0.002 & 0.003 \end{bmatrix}$	$\begin{bmatrix} -0.004 & 0.002 & 0.002 & -0.005 \\ -0.007 & 0.004 & -0.001 & 0.007 \\ 0.002 & 0.002 & 0.003 & 0.011 \end{bmatrix}$	$\begin{bmatrix} -0.002 & 0.001 & 0.001 \\ 0.001 & -0.003 & -0.003 \\ 0.001 & -0.003 & -0.003 \end{bmatrix}$	$\begin{bmatrix} -0.001 & -0.002 & 0.002 & 0.000 \\ -0.002 & 0.003 & -0.002 & 0.001 \\ 0.002 & -0.002 & 0.002 & -0.001 \\ 0.000 & 0.001 & -0.001 & -0.001 \end{bmatrix}$
		MSE	$\begin{bmatrix} 0.003 & 0.002 & 0.002 & 0.003 \\ 0.003 & 0.003 & 0.003 & 0.003 \\ 0.002 & 0.003 & 0.002 & 0.003 \end{bmatrix}$	$\begin{bmatrix} 0.003 & 0.003 & 0.002 & 0.002 \\ 0.004 & 0.003 & 0.002 & 0.004 \\ 0.002 & 0.002 & 0.002 & 0.007 \end{bmatrix}$	$\begin{bmatrix} 0.001 & 0.001 & 0.001 \\ 0.001 & 0.001 & 0.001 \\ 0.001 & 0.001 & 0.001 \end{bmatrix}$	$\begin{bmatrix} 0.001 & 0.001 & 0.001 & 0.001 \\ 0.001 & 0.001 & 0.001 & 0.001 \\ 0.001 & 0.001 & 0.001 & 0.001 \\ 0.001 & 0.001 & 0.001 & 0.001 \end{bmatrix}$
MVMMNW	100	Bias	$\begin{bmatrix} 0.005 & -0.010 & 0.001 & 0.004 \\ 0.008 & 0.001 & -0.003 & -0.015 \\ 0.011 & -0.007 & 0.004 & -0.021 \end{bmatrix}$	$\begin{bmatrix} -0.006 & 0.014 & -0.007 & -0.006 \\ -0.009 & 0.003 & 0.005 & 0.022 \\ -0.006 & 0.012 & -0.003 & 0.028 \end{bmatrix}$	$\begin{bmatrix} -0.023 & 0.011 & -0.004 \\ 0.011 & -0.023 & -0.014 \\ -0.004 & -0.014 & -0.030 \end{bmatrix}$	$\begin{bmatrix} 0.002 & -0.001 & 0.003 & -0.001 \\ -0.001 & 0.006 & -0.004 & 0.004 \\ 0.003 & -0.004 & 0.003 & -0.002 \\ -0.001 & 0.004 & -0.002 & 0.003 \end{bmatrix}$
		MSE	$\begin{bmatrix} 0.053 & 0.056 & 0.050 & 0.056 \\ 0.062 & 0.055 & 0.050 & 0.067 \\ 0.053 & 0.058 & 0.055 & 0.085 \end{bmatrix}$	$\begin{bmatrix} 0.056 & 0.061 & 0.051 & 0.058 \\ 0.071 & 0.058 & 0.051 & 0.073 \\ 0.057 & 0.063 & 0.055 & 0.104 \end{bmatrix}$	$\begin{bmatrix} 0.006 & 0.004 & 0.003 \\ 0.004 & 0.006 & 0.004 \\ 0.003 & 0.004 & 0.007 \end{bmatrix}$	$\begin{bmatrix} 0.007 & 0.005 & 0.004 & 0.005 \\ 0.005 & 0.006 & 0.005 & 0.005 \\ 0.004 & 0.005 & 0.006 & 0.004 \\ 0.005 & 0.005 & 0.004 & 0.008 \end{bmatrix}$
	500	Bias	$\begin{bmatrix} -0.004 & 0.001 & 0.001 & 0.002 \\ 0.003 & 0.000 & -0.001 & -0.006 \\ 0.001 & -0.001 & 0.002 & -0.003 \end{bmatrix}$	$\begin{bmatrix} 0.002 & 0.004 & -0.003 & -0.006 \\ -0.007 & 0.001 & 0.003 & 0.010 \\ 0.000 & 0.002 & -0.003 & 0.006 \end{bmatrix}$	$\begin{bmatrix} -0.007 & 0.003 & 0.000 \\ 0.003 & -0.004 & -0.002 \\ 0.000 & -0.002 & -0.005 \end{bmatrix}$	$\begin{bmatrix} 0.002 & -0.001 & 0.002 & -0.001 \\ -0.001 & 0.001 & -0.001 & 0.001 \\ 0.002 & -0.001 & 0.000 & -0.002 \\ -0.001 & 0.001 & -0.002 & 0.000 \end{bmatrix}$
		MSE	$\begin{bmatrix} 0.011 & 0.011 & 0.01 & 0.011 \\ 0.013 & 0.011 & 0.01 & 0.013 \\ 0.010 & 0.011 & 0.01 & 0.017 \end{bmatrix}$	$\begin{bmatrix} 0.012 & 0.011 & 0.01 & 0.012 \\ 0.014 & 0.011 & 0.01 & 0.014 \\ 0.010 & 0.012 & 0.01 & 0.020 \end{bmatrix}$	$\begin{bmatrix} 0.001 & 0.001 & 0.001 \\ 0.001 & 0.001 & 0.001 \\ 0.001 & 0.001 & 0.001 \end{bmatrix}$	$\begin{bmatrix} 0.001 & 0.001 & 0.001 & 0.001 \\ 0.001 & 0.001 & 0.001 & 0.001 \\ 0.001 & 0.001 & 0.001 & 0.001 \\ 0.001 & 0.001 & 0.001 & 0.001 \end{bmatrix}$

7 Conclusion

This paper has introduced a new family of matrix variate distributions whose component pdfs arise from the mean-mixture of matrix variate normal model. Some properties and characteristics as well as three special cases of the new model are derived. We have developed a computationally EM-based algorithm for calibrating the matrix type parameters to the data. It is shown that the MVMMN distribution is closed under the formation of marginal and conditional distributions and under affine transformation which make it flexible to use in the various fields of three-variate data analysis, such as multivariate time series, image processing and longitudinal data analysis. Simulation results show that the ML estimates obtained via the ECM algorithm are empirically consistent. Moreover, numerical results from application to real dataset reveal that the proposed model is well suited in dealing with the skewed matrix variate experimental data.

The utility of our current approach can be extended to accommodate censored data based on a recent work studied in the multivariate case by [31, 32]. It may also be interesting to propose a family of scale mixture of MVMMN distribution to deal with heavy tailed three-way data. Another possible extension of the work herein is to consider finite mixture model based on the MVMMN distribution as a promising tools in

Table 4. Parameters estimates and the performance summary of three matrix models on the LSD subsets.

Dataset	Parameter	red soil	cotton crop
MVRSN	$\mathbf{M}$	$\begin{bmatrix} 54.50 & 53.70 & 53.88 & 53.63 & 53.39 & 53.75 & 53.64 & 53.88 & 54.03 \\ 76.02 & 75.05 & 75.05 & 74.40 & 73.96 & 74.09 & 73.97 & 73.95 & 74.11 \\ 91.92 & 91.21 & 91.46 & 90.98 & 89.88 & 90.31 & 90.76 & 90.44 & 89.65 \\ 77.10 & 76.85 & 77.29 & 76.28 & 75.78 & 76.27 & 76.65 & 76.13 & 76.04 \end{bmatrix}$	$\begin{bmatrix} 46.00 & 42.45 & 40.70 & 48.54 & 45.92 & 43.23 & 50.43 & 49.26 & 47.54 \\ 34.02 & 28.35 & 25.29 & 38.63 & 33.85 & 29.33 & 42.76 & 40.14 & 36.56 \\ 115.13 & 116.99 & 116.38 & 113.82 & 115.33 & 116.39 & 111.31 & 112.05 & 112.91 \\ 123.46 & 128.03 & 127.46 & 119.52 & 124.22 & 126.47 & 114.94 & 116.70 & 119.61 \end{bmatrix}$
	$\mathbf{\Lambda}$	$\begin{bmatrix} 11.01 & 11.62 & 10.98 & 11.51 & 11.46 & 10.70 & 10.77 & 10.32 & 10.24 \\ 22.97 & 23.97 & 23.05 & 24.49 & 25.17 & 24.09 & 23.99 & 24.43 & 23.84 \\ 18.88 & 20.09 & 18.97 & 19.92 & 21.24 & 20.27 & 19.70 & 20.68 & 20.95 \\ 13.43 & 13.87 & 13.07 & 14.44 & 15.15 & 14.20 & 13.97 & 14.58 & 14.13 \end{bmatrix}$	$\begin{bmatrix} 4.28 & 8.62 & 11.76 & 0.46 & 3.43 & 7.05 & -0.90 & 0.13 & 2.52 \\ 8.52 & 16.03 & 21.88 & 1.49 & 6.46 & 13.16 & -2.07 & 0.43 & 5.53 \\ -1.98 & -3.77 & -3.37 & 0.36 & -1.19 & -1.98 & 2.68 & 1.78 & 0.47 \\ -7.44 & -12.73 & -13.18 & -1.24 & -6.06 & -9.47 & 3.27 & 1.34 & -3.06 \end{bmatrix}$
	$\mathbf{\Sigma}$	$\begin{bmatrix} 10.12 & 8.07 & 5.32 & 3.97 \\ 8.07 & 26.56 & 16.33 & 10.51 \\ 5.32 & 16.33 & 23.70 & 11.04 \\ 3.97 & 10.51 & 11.04 & 12.46 \end{bmatrix}$	$\begin{bmatrix} 17.43 & 24.37 & -13.00 & -27.12 \\ 24.37 & 50.79 & -25.58 & -53.50 \\ -13.00 & -25.58 & 44.79 & 53.03 \\ -27.12 & -53.50 & 53.03 & 98.70 \end{bmatrix}$
	$\mathbf{\Psi}$	$\begin{bmatrix} 2.16 & 1.36 & 0.76 & 1.12 & 0.80 & 0.43 & 0.66 & 0.46 & 0.22 \\ 1.36 & 2.03 & 1.39 & 0.84 & 0.88 & 0.89 & 0.43 & 0.45 & 0.45 \\ 0.76 & 1.39 & 2.30 & 0.56 & 0.81 & 1.30 & 0.26 & 0.46 & 0.74 \\ 1.12 & 0.84 & 0.56 & 1.95 & 0.99 & 0.49 & 1.21 & 0.90 & 0.47 \\ 0.80 & 0.88 & 0.81 & 0.99 & 1.51 & 0.97 & 0.69 & 0.77 & 0.72 \\ 0.43 & 0.89 & 1.30 & 0.49 & 0.97 & 2.07 & 0.45 & 0.72 & 1.15 \\ 0.66 & 0.43 & 0.26 & 1.21 & 0.69 & 0.45 & 2.53 & 1.44 & 0.72 \\ 0.46 & 0.45 & 0.46 & 0.90 & 0.77 & 0.72 & 1.44 & 1.86 & 1.12 \\ 0.22 & 0.45 & 0.74 & 0.47 & 0.72 & 1.15 & 0.72 & 1.12 & 1.91 \end{bmatrix}$	$\begin{bmatrix} 2.78 & 1.88 & 1.31 & 2.00 & 1.60 & 0.97 & 1.35 & 1.22 & 0.97 \\ 1.88 & 2.18 & 1.62 & 1.47 & 1.35 & 1.12 & 0.96 & 0.81 & 0.79 \\ 1.31 & 1.62 & 2.20 & 1.18 & 1.06 & 1.11 & 0.83 & 0.69 & 0.64 \\ 2.00 & 1.47 & 1.18 & 2.71 & 1.80 & 1.08 & 2.06 & 1.83 & 1.32 \\ 1.60 & 1.35 & 1.06 & 1.80 & 2.14 & 1.45 & 1.51 & 1.59 & 1.54 \\ 0.97 & 1.12 & 1.11 & 1.08 & 1.45 & 1.96 & 1.22 & 1.31 & 1.53 \\ 1.35 & 0.96 & 0.83 & 2.06 & 1.51 & 1.22 & 3.23 & 2.44 & 1.81 \\ 1.22 & 0.81 & 0.69 & 1.83 & 1.59 & 1.31 & 2.44 & 2.83 & 2.19 \\ 0.97 & 0.79 & 0.64 & 1.32 & 1.54 & 1.53 & 1.81 & 2.19 & 2.76 \end{bmatrix}$
MVMMNE	$\mathbf{M}$	$\begin{bmatrix} 55.37 & 54.60 & 54.73 & 54.53 & 54.28 & 54.58 & 54.48 & 54.69 & 54.81 \\ 77.59 & 76.71 & 76.64 & 76.07 & 75.70 & 75.73 & 75.58 & 75.60 & 75.71 \\ 93.17 & 92.58 & 92.79 & 92.33 & 91.34 & 91.72 & 92.10 & 91.85 & 91.06 \\ 77.99 & 77.82 & 78.22 & 77.26 & 76.82 & 77.26 & 77.62 & 77.13 & 76.99 \end{bmatrix}$	$\begin{bmatrix} 46.82 & 44.03 & 42.84 & 48.46 & 46.39 & 44.35 & 49.94 & 48.95 & 47.67 \\ 35.60 & 31.34 & 29.34 & 38.63 & 34.79 & 31.45 & 41.74 & 39.62 & 37.01 \\ 114.94 & 116.50 & 115.98 & 114.24 & 115.51 & 116.47 & 112.39 & 112.94 & 113.59 \\ 122.24 & 125.79 & 125.21 & 119.77 & 123.59 & 125.29 & 116.45 & 117.85 & 119.93 \end{bmatrix}$
	$\mathbf{\Lambda}$	$\begin{bmatrix} 8.68 & 9.19 & 8.69 & 9.09 & 9.06 & 8.47 & 8.51 & 8.16 & 8.11 \\ 18.38 & 19.16 & 18.43 & 19.59 & 20.12 & 19.29 & 19.22 & 19.57 & 19.10 \\ 15.14 & 16.08 & 15.14 & 15.94 & 16.99 & 16.19 & 15.77 & 16.56 & 16.78 \\ 10.77 & 11.08 & 10.42 & 11.56 & 12.12 & 11.34 & 11.16 & 11.66 & 11.32 \end{bmatrix}$	$\begin{bmatrix} 2.26 & 4.61 & 6.29 & 0.41 & 1.99 & 3.93 & -0.15 & 0.40 & 1.68 \\ 4.53 & 8.51 & 11.64 & 1.06 & 3.70 & 7.32 & -0.46 & 0.83 & 3.51 \\ -1.23 & -2.21 & -2.02 & -0.16 & -1.03 & -1.50 & 0.84 & 0.38 & -0.34 \\ -4.12 & -6.89 & -7.20 & -1.14 & -3.72 & -5.61 & 0.84 & -0.19 & -2.51 \end{bmatrix}$
	$\mathbf{\Sigma}$	$\begin{bmatrix} 10.55 & 8.53 & 5.66 & 4.22 \\ 8.53 & 27.63 & 17.04 & 10.98 \\ 5.66 & 17.04 & 24.61 & 11.50 \\ 4.22 & 10.98 & 11.50 & 12.93 \end{bmatrix}$	$\begin{bmatrix} 18.13 & 25.28 & -13.55 & -28.27 \\ 25.28 & 52.87 & -26.70 & -55.89 \\ -13.55 & -26.70 & 47.29 & 55.88 \\ -28.27 & -55.89 & 55.88 & 103.84 \end{bmatrix}$
	$\mathbf{\Psi}$	$\begin{bmatrix} 2.11 & 1.34 & 0.77 & 1.11 & 0.80 & 0.44 & 0.67 & 0.47 & 0.24 \\ 1.34 & 2.00 & 1.37 & 0.85 & 0.89 & 0.90 & 0.45 & 0.47 & 0.46 \\ 0.77 & 1.37 & 2.25 & 0.57 & 0.82 & 1.29 & 0.28 & 0.48 & 0.74 \\ 1.11 & 0.85 & 0.57 & 1.91 & 0.99 & 0.50 & 1.20 & 0.90 & 0.48 \\ 0.80 & 0.89 & 0.82 & 0.99 & 1.49 & 0.97 & 0.70 & 0.77 & 0.73 \\ 0.44 & 0.90 & 1.29 & 0.50 & 0.97 & 2.04 & 0.47 & 0.72 & 1.14 \\ 0.67 & 0.45 & 0.28 & 1.20 & 0.70 & 0.47 & 2.47 & 1.41 & 0.72 \\ 0.47 & 0.47 & 0.48 & 0.90 & 0.77 & 0.72 & 1.41 & 1.82 & 1.11 \\ 0.24 & 0.46 & 0.74 & 0.48 & 0.73 & 1.14 & 0.72 & 1.11 & 1.87 \end{bmatrix}$	$\begin{bmatrix} 2.65 & 1.78 & 1.24 & 1.90 & 1.51 & 0.91 & 1.27 & 1.15 & 0.90 \\ 1.78 & 2.05 & 1.51 & 1.39 & 1.26 & 1.02 & 0.88 & 0.74 & 0.71 \\ 1.24 & 1.51 & 2.04 & 1.10 & 0.98 & 1.00 & 0.74 & 0.61 & 0.56 \\ 1.90 & 1.39 & 1.10 & 2.58 & 1.71 & 1.02 & 1.97 & 1.74 & 1.25 \\ 1.51 & 1.26 & 0.98 & 1.71 & 2.02 & 1.35 & 1.43 & 1.51 & 1.44 \\ 0.91 & 1.02 & 1.00 & 1.02 & 1.35 & 1.82 & 1.13 & 1.22 & 1.42 \\ 1.27 & 0.88 & 0.74 & 1.97 & 1.43 & 1.13 & 3.09 & 2.33 & 1.72 \\ 1.15 & 0.74 & 0.61 & 1.74 & 1.51 & 1.22 & 2.33 & 2.70 & 2.08 \\ 0.90 & 0.71 & 0.56 & 1.25 & 1.44 & 1.42 & 1.72 & 2.08 & 2.61 \end{bmatrix}$
MVMMNW	$\mathbf{M}$	$\begin{bmatrix} 51.52 & 50.57 & 50.93 & 50.53 & 50.32 & 50.89 & 50.74 & 51.09 & 51.27 \\ 70.14 & 68.86 & 69.10 & 68.14 & 67.51 & 67.91 & 67.93 & 67.72 & 68.00 \\ 87.15 & 86.03 & 86.52 & 85.90 & 84.42 & 85.04 & 85.75 & 85.13 & 84.26 \\ 73.70 & 73.23 & 73.86 & 72.62 & 71.90 & 72.58 & 73.06 & 72.37 & 72.40 \end{bmatrix}$	$\begin{bmatrix} 45.01 & 39.48 & 36.28 & 49.35 & 45.19 & 40.75 & 51.86 & 50.09 & 47.16 \\ 32.00 & 22.74 & 17.18 & 39.81 & 32.33 & 24.59 & 45.62 & 41.55 & 35.42 \\ 114.99 & 117.62 & 116.95 & 112.69 & 114.92 & 116.43 & 109.29 & 110.53 & 111.96 \\ 124.78 & 131.89 & 131.66 & 118.19 & 125.29 & 129.24 & 111.68 & 114.63 & 119.67 \end{bmatrix}$
	$\mathbf{\Lambda}$	$\begin{bmatrix} 13.21 & 13.93 & 13.16 & 13.79 & 13.73 & 12.81 & 12.91 & 12.39 & 12.27 \\ 27.21 & 28.46 & 27.37 & 29.00 & 29.83 & 28.56 & 28.32 & 28.93 & 28.26 \\ 22.30 & 23.84 & 22.57 & 23.58 & 25.19 & 24.10 & 23.31 & 24.52 & 24.85 \\ 15.88 & 16.51 & 15.57 & 17.08 & 17.95 & 16.88 & 16.57 & 17.31 & 16.76 \end{bmatrix}$	$\begin{bmatrix} 4.77 & 10.74 & 15.06 & -0.57 & 3.74 & 8.84 & -2.43 & -0.86 & 2.57 \\ 9.54 & 20.06 & 27.91 & -0.13 & 7.21 & 16.62 & -5.09 & -1.29 & 5.99 \\ -1.51 & -3.90 & -3.50 & 1.62 & -0.51 & -1.71 & 4.62 & 3.27 & 1.51 \\ -7.81 & -15.23 & -16.00 & 0.52 & -6.36 & -11.22 & 6.57 & 3.55 & -2.64 \end{bmatrix}$
	$\mathbf{\Sigma}$	$\begin{bmatrix} 10.04 & 7.89 & 5.17 & 3.86 \\ 7.89 & 26.42 & 16.21 & 10.42 \\ 5.17 & 16.21 & 23.60 & 10.97 \\ 3.86 & 10.42 & 10.97 & 12.41 \end{bmatrix}$	$\begin{bmatrix} 17.55 & 24.60 & -13.16 & -27.40 \\ 24.60 & 51.26 & -25.85 & -54.03 \\ -13.16 & -25.85 & 44.80 & 53.22 \\ -27.40 & -54.03 & 53.22 & 99.23 \end{bmatrix}$
	$\mathbf{\Psi}$	$\begin{bmatrix} 2.14 & 1.34 & 0.73 & 1.10 & 0.77 & 0.40 & 0.64 & 0.43 & 0.19 \\ 1.34 & 2.00 & 1.36 & 0.82 & 0.85 & 0.86 & 0.41 & 0.42 & 0.42 \\ 0.73 & 1.36 & 2.27 & 0.53 & 0.78 & 1.27 & 0.23 & 0.44 & 0.71 \\ 1.10 & 0.82 & 0.53 & 1.93 & 0.97 & 0.46 & 1.19 & 0.88 & 0.44 \\ 0.77 & 0.85 & 0.78 & 0.97 & 1.48 & 0.95 & 0.67 & 0.74 & 0.70 \\ 0.40 & 0.86 & 1.27 & 0.46 & 0.95 & 2.05 & 0.43 & 0.69 & 1.13 \\ 0.64 & 0.41 & 0.23 & 1.19 & 0.67 & 0.43 & 2.52 & 1.42 & 0.70 \\ 0.43 & 0.42 & 0.44 & 0.88 & 0.74 & 0.69 & 1.42 & 1.83 & 1.10 \\ 0.19 & 0.42 & 0.71 & 0.44 & 0.70 & 1.13 & 0.70 & 1.10 & 1.89 \end{bmatrix}$	$\begin{bmatrix} 2.82 & 1.94 & 1.39 & 2.02 & 1.63 & 1.02 & 1.37 & 1.24 & 1.00 \\ 1.94 & 2.24 & 1.69 & 1.53 & 1.39 & 1.16 & 1.02 & 0.86 & 0.83 \\ 1.39 & 1.69 & 2.28 & 1.26 & 1.12 & 1.16 & 0.91 & 0.77 & 0.71 \\ 2.02 & 1.53 & 1.26 & 2.70 & 1.82 & 1.13 & 2.05 & 1.82 & 1.34 \\ 1.63 & 1.39 & 1.12 & 1.82 & 2.16 & 1.48 & 1.52 & 1.61 & 1.56 \\ 1.02 & 1.16 & 1.16 & 1.13 & 1.48 & 1.99 & 1.26 & 1.35 & 1.56 \\ 1.37 & 1.02 & 0.91 & 2.05 & 1.52 & 1.26 & 3.19 & 2.42 & 1.81 \\ 1.24 & 0.86 & 0.77 & 1.82 & 1.61 & 1.35 & 2.42 & 2.82 & 2.20 \\ 1.00 & 0.83 & 0.71 & 1.34 & 1.56 & 1.56 & 1.81 & 2.20 & 2.78 \end{bmatrix}$

		red soil			cotton crop		
Model	Criterion →	$\ell_{\max}$	AIC	BIC	$\ell_{\max}$	AIC	BIC
RMVSN		-46110.78	92475.55	93000.49	-24169.20	48592.41	49025.69
MVMMNE		-46167.80	92589.60	93114.54	**-24137.68**	**48529.37**	**48962.65**
MVMMNW		**-46079.34**	**92412.68**	**92937.62**	-24183.09	48620.18	49053.46

classification and clustering heterogeneous matrix-valued asymmetric data [19, 33]. It would be of interest the distributions of the associated eigenvalues of the quadratic form (Theorem 8; for the complex form) to compute the channel capacity in wireless communication systems, since experimental data do not follow necessarily a normal distribution (see [34, 35]).

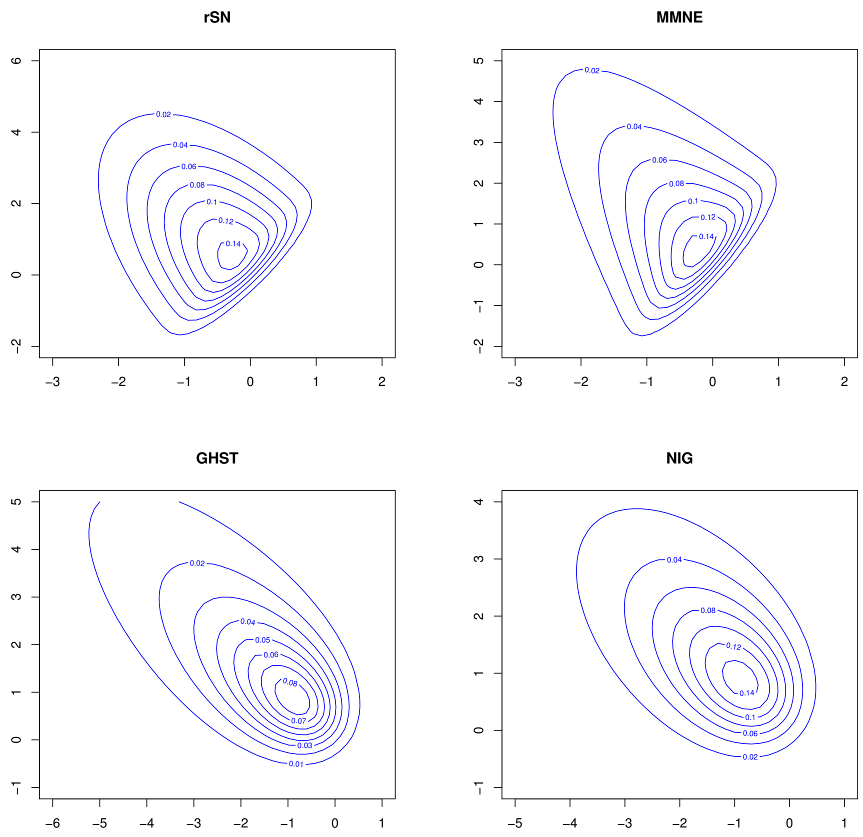

Fig 2. Contour plots comparison of special cases of the MMN and MVMN families.

Acknowledgments

M. Naderi and A. Bekker acknowledge the research support provided by the National Research Foundation (NRF) of South Africa, Reference: CPRR160403161466 grant Number: 105840 and STATOMET. M. Arashi is also based upon research supported in part by the NRF of South Africa, Ref: IFR170227223754 grant Number: 109214 and SARChI Research Chair-UID: 71199. All computations were carried out by R language and the computer program is available from the first author upon request.

Appendix A: Comparison of contour plots of the MMN and MVMN families

Figure 2 illustrate the contour plots of the bivariate rSN and bivariate exponentiated MMN (MMNE) distributions as special cases of MMN family as well as the contour plots of the bivariate generalized hyperbolic skew-t (GHST) and bivariate normal inverse Gaussian (NIG) distributions as special cases of MVMN family.

References

1. Azzalini A. A class of distributions which includes the normal ones. Scandinavian Journal of Statistics. 1985;12(2):171–178.

2. Azzalini A, Genton MG. Robust likelihood methods based on the skew-t and related distributions. International Statistical Review. 2008;76(1):106–129.

3. Pyne S, Hu X, Wang K, Rossin E, Lin TI, Maier LM, et al. Automated high-dimensional flow cytometric data analysis. Proceedings of the National Academy of Sciences. 2009;106(21):8519–8524.

4. Lin TI. Robust mixture modeling using multivariate skew t distributions. Statistics and Computing. 2009;20(3):343–356.

5. Cabral CRB, Lachos VH, Prates MO. Multivariate mixture modeling using skew-normal independent distributions. Computational Statistics & Data Analysis. 2012;56(1):126–142.

6. Negarestani H, Jamalizadeh A, Shafiei S, Balakrishnan N. Mean mixtures of normal distributions: properties, inference and application. Metrika. 2019;82(4):501–528.

7. Ho HJ, Pyne S, Lin TI. Maximum likelihood inference for mixtures of skew Student-t-normal distributions through practical EM-type algorithms. Statistics and Computing. 2012;22(1):287–299.

8. McNeil AJ, Frey R, Embrechts P. Quantitative risk management: Concepts, techniques and tools. 2005;.

9. Roy SN. Some aspects of multivariate analysis. Statistical Publishing Society, Kolkata; 1957.

10. Girko V, Gupta A. Multivariate elliptically contoured linear models and some aspects of the theory of random matrices. In: Multidimensional Statistical Analysis and Theory of Random Matrices: Proceedings of the Sixth Eugene Lukacs Symposium, Bowling Green, Ohio, USA, 29–30 March 1996. Walter de Gruyter GmbH & Co KG; 1996. p. 327.

11. Anderlucci L, Viroli C. Covariance pattern mixture models for the analysis of multivariate heterogeneous longitudinal data. The Annals of Applied Statistics. 2015;9(2):777–800.

12. Kshirsagar AM, Bartlett MS. Some Extensions of the Multivariate t-Distribution and the Multivariate Generalization of the Distribution of the Regression Coefficient. Mathematical Proceedings of the Cambridge Philosophical Society. 1961;57(01):80.

13. Bulut YM, Arslan O. Matrix variate slash distribution. Journal of Multivariate Analysis. 2015;137:173–178.

14. Chen JT, Gupta AK. Matrix variate skew normal distributions. Statistics. 2005;39(3):247–253.

15. Harrar SW, Gupta AK. On matrix variate skew-normal distributions. Statistics. 2008;42(2):179–194.

16. Akdemir D, Gupta AK. A matrix variate skew distribution. European Journal of Pure and Applied Mathematics. 2010;3(2):128–140.

17. Zheng S, Hardin JM, Gupta AK. The inverse problem of multivariate and matrix-variate skew normal distributions. Statistics. 2012;46(3):361–371.

18. Dempster AP, Laird NM, Rubin DB. Maximum likelihood from incomplete data via the EM algorithm. Journal of the royal statistical society Series B (methodological). 1977;39(1):1–38.

19. Gallaugher MPB, McNicholas PD. Finite mixtures of skewed matrix variate distributions. Pattern Recognition. 2018;80:83–93.

20. Gallaugher MPB, McNicholas PD. Three skewed matrix variate distributions. Statistics & Probability Letters. 2019;145:103–109.

21. Boyd S, Vandenberghe L. Convex optimization. Cambridge University Press; 2004.

22. Gupta AK, Nagar DK. Matrix variate distributions. Chapman and Hall/CRC; 1999.

23. Singull M, Koski T. On the Distribution of Matrix Quadratic Forms. Communications in Statistics - Theory and Methods. 2012;41(18):3403–3415.

24. Meng XL, Rubin DB. Maximum likelihood estimation via the ECM algorithm: A general framework. Biometrika. 1993;80(2):267–278.

25. Aitken AC. On Bernoulli's Numerical Solution of Algebraic Equations. Proceedings of the Royal Society of Edinburgh. 1927;46:289–305.

26. Böhning D, Dietz E, Schaub R, Schlattmann P, Lindsay BG. The distribution of the likelihood ratio for mixtures of densities from the one-parameter exponential family. Annals of the Institute of Statistical Mathematics. 1994;46(2):373–388.

27. Lindsay BG. Mixture models: theory, geometry and applications. In: NSF-CBMS regional conference series in probability and statistics. JSTOR; 1995. p. i–163.

28. Akaike H. Information theory and an extension of the maximum likelihood principle. In: Selected papers of hirotugu akaike. Springer; 1998. p. 199–213.

29. Schwarz G, et al. Estimating the dimension of a model. The annals of statistics. 1978;6(2):461–464.

30. Good IJ. The population frequencies of species and the estimation of population parameters. Biometrika. 1953;40:237–260.

31. Wang WL, Lin TI, Lachos VH. Extending multivariate-t linear mixed models for multiple longitudinal data with censored responses and heavy tails. Statistical Methods in Medical Research. 2015;27(1):48–64.

32. Lin TI, Lachos VH, Wang WL. Multivariate longitudinal data analysis with censored and intermittent missing responses. Statistics in Medicine. 2018;37(19):2822–2835.

33. Melnykov V, Zhu X. On model-based clustering of skewed matrix data. Journal of Multivariate Analysis. 2018;167:181–194.

34. Ratnarajah T, Vaillancourt R. Quadratic Forms on Complex Random Matrices and Multiple-Antenna Systems. IEEE Transactions on Information Theory. 2005;51(8):2976–2984.

35. Bekker A, Ferreira J. Bivariate gamma type distributions for modeling wireless performance metrics. Statistics, Optimization & Information Computing. 2018;6(3).
