# Supplementary material for: A theoretical framework for Landsat data modeling based on the matrix variate mean-mixture of normal model
Source: PLoS One. 2020 Apr 9;15(4):e0230773. doi: 10.1371/journal.pone.0230773 (PMC7144982; doi:10.1371/journal.pone.0230773)
Supplement: Attachment — Submitted filename: Response to Reviewers.pdf [file pone.0230773.s002.pdf]

The authors' responses to reviewer comments *(in italics)* **on PONE-D-19-24001**

A theoretical framework for Landsat data modeling based on the matrix variate mean-mixture of normal model

January 25, 2020

Additional Editor Comments

Dear author,

we are now sending you the results of the reviewing by one referee. The remaing ones never materialized, so we decided, since it pointed minor revision, to proceed with this only one. Please, improve the theoretical framework of the process putting most of the things in an easier way. Please, add coments clarifying each of the mathematical steps allowing future readers to better undertanding your intentions. As suggested by the reviewer, try to put auxiliar figures and schemes like flowcharts.

Response:

We thank the Associate Editor (AE) for providing very encouraging comments. In the revised manuscript, we have described each query of the reviewer. Our point-by-point responses done to clarify all comments and all suggested lines are as follows.

Reviewer 1's comments:

General comments:

The author's research is valuable and significant. In this paper, authors show a strong foundation in mathematics and physics, mathematical logic is rigorous. This research is innovative, however, the theoretical process would be easier to understand if more graphics were used.

Response:

We highly appreciate the reviewer for encouraging words and nice suggestions. The paper has been revised along the lines suggested.

Specific comments:

1) *Proposed family: please establish the necessary image summary for the derivation of the following formula.*

Response:

To clarify this points, we have added the following sentences:

"To start the whole process, we begin with some notations and definitions. A random matrix variable $\boldsymbol{\mathcal{X}} \in \mathbb{R}^{p\times n}$ defined as

$$\boldsymbol{\mathcal{X}} = \begin{pmatrix} X_{11} & \cdots & X_{1n} \\ \vdots & \ddots & \vdots \\ X_{p1} & \cdots & X_{pn} \end{pmatrix},$$

follows a MVN distribution if its pdf is given as

$$\phi_{p,n}(\mathbf{X}; \mathbf{M}, \boldsymbol{\Sigma}, \boldsymbol{\Psi}) = \frac{1}{(2\pi)^{(\frac{np}{2})}|\boldsymbol{\Sigma}|^{\frac{n}{2}}|\boldsymbol{\Psi}|^{\frac{p}{2}}} \operatorname{etr}\left\{-\frac{1}{2}\boldsymbol{\delta}(\mathbf{X}, \mathbf{M}, \boldsymbol{\Psi}, \boldsymbol{\Sigma})\right\}, \tag{3}$$

where $\operatorname{etr}\{A\} = \exp(\operatorname{tr}(A))$, $\operatorname{tr}(\cdot)$ is the trace operator of a matrix, $\boldsymbol{\delta}(\mathbf{X}, \mathbf{M}, \boldsymbol{\Psi}, \boldsymbol{\Sigma}) = \boldsymbol{\Sigma}^{-1}\big(\mathbf{X} - \mathbf{M}\big)\boldsymbol{\Psi}^{-1}\big(\mathbf{X} - \mathbf{M}\big)^{\top}$ denotes the matrix variate Mahalanobis squared distance, and the mean matrix $\mathbf{M}$ and two dispersion matrices $\boldsymbol{\Sigma} \in \mathbb{R}^{p\times p}$, $\boldsymbol{\Psi} \in \mathbb{R}^{n\times n}$ are defined as

$$\mathbf{M} = \begin{pmatrix} \mu_{11} & \cdots & \mu_{1n} \\ \vdots & \ddots & \vdots \\ \mu_{p1} & \cdots & \mu_{pn} \end{pmatrix}, \ \boldsymbol{\Sigma} = \begin{pmatrix} \sigma_{11} & \cdots & \sigma_{1p} \\ \vdots & \ddots & \vdots \\ \sigma_{p1} & \cdots & \sigma_{pp} \end{pmatrix}, \ \boldsymbol{\Psi} = \begin{pmatrix} \psi_{11} & \cdots & \psi_{1n} \\ \vdots & \ddots & \vdots \\ \psi_{n1} & \cdots & \psi_{nn} \end{pmatrix}.$$

We shall use notation $\boldsymbol{\mathcal{X}} \sim \mathcal{N}_{p,n}(\mathbf{M}, \boldsymbol{\Sigma}, \boldsymbol{\Psi})$ if $\boldsymbol{\mathcal{X}}$ has pdf (3). The following definition is a new result from the representation (1) in the matrix format.

Definition 1 *A random matrix variable $\boldsymbol{\mathcal{Y}}$ is said to have a MVMMN distribution if it can be generated by the stochastic representation*

$$\boldsymbol{\mathcal{Y}} \stackrel{d}{=} \boldsymbol{M} + W\boldsymbol{\Lambda} + \boldsymbol{\mathcal{X}}, \tag{4}$$

where $\boldsymbol{\mathcal{X}} \sim \mathcal{N}_{p,n}(\mathbf{0}, \boldsymbol{\Sigma}, \boldsymbol{\Psi})$, W is a random variable, independent of $\boldsymbol{\mathcal{X}}$, distributed by $h(w; \boldsymbol{\nu})$, and $\boldsymbol{\Lambda} \in \mathbb{R}^{p\times n}$ is a skewness matrix defined as

$$\boldsymbol{\Lambda} = \begin{pmatrix} \lambda_{11} & \cdots & \lambda_{1n} \\ \vdots & \ddots & \vdots \\ \lambda_{p1} & \cdots & \lambda_{pn} \end{pmatrix}.$$

It can be easily seen that the hierarchical representation of MVMMN model is

$$\begin{aligned} \boldsymbol{\mathcal{Y}}|W = w &\sim \mathcal{N}_{p,n}(\boldsymbol{M} + w\boldsymbol{\Lambda}, \boldsymbol{\Sigma}, \boldsymbol{\Psi}), \\ W &\sim h(w; \boldsymbol{\nu}). \end{aligned} \tag{5}$$

Hence, the pdf of $\boldsymbol{\mathcal{Y}} \sim \mathcal{MVMMN}_{p,n}(\boldsymbol{M}, \boldsymbol{\Lambda}, \boldsymbol{\Sigma}, \boldsymbol{\Psi}, W)$ can be given as

$$f(\boldsymbol{Y}; \boldsymbol{M}, \boldsymbol{\Lambda}, \boldsymbol{\Sigma}, \boldsymbol{\Psi}, \boldsymbol{\nu}) = \int_{-\infty}^{\infty} \phi_{p,n}(\boldsymbol{Y}; \boldsymbol{M} + w\boldsymbol{\Lambda}, \boldsymbol{\Sigma}, \boldsymbol{\Psi}) h(w; \boldsymbol{\nu})\, dw, \qquad \boldsymbol{Y} \in \mathbb{R}^{p\times n}." \tag{6}$$

2) *Theorem 4: please describe the proof process in detail.*

Response:

The proof of theorem has been revised as bellow.

"Based on theorem 2.3.1 of [22], we have

$$\boldsymbol{\mathcal{X}} \sim \mathcal{N}_{p,n}(\mathbf{M}, \mathbf{\Sigma}, \mathbf{\Psi}) \leftrightarrow \boldsymbol{\mathcal{X}}^\top \sim \mathcal{N}_{p,n}(\mathbf{M}^\top, \mathbf{\Psi}, \mathbf{\Sigma}).$$

Now, applying this transpose property of the MVN distribution into the hierarchical representation (5) results in

$$\boldsymbol{\mathcal{Y}}|W = w \sim \mathcal{N}_{p,n}(\mathbf{M} + w\mathbf{\Lambda}, \mathbf{\Sigma}, \mathbf{\Psi}) \leftrightarrow \boldsymbol{\mathcal{Y}}^\top|W = w \sim \mathcal{N}_{p,n}(\mathbf{M}^\top + w\mathbf{\Lambda}^\top, \mathbf{\Psi}, \mathbf{\Sigma}).''$$

3) *Theorem 8: please supplement this missing proof process.*

Response:

The proof has been revised as follows.

"Using hierarchical representation (5) of the MVMMN model, we have $\boldsymbol{\mathcal{Y}}|W = w \sim \mathcal{N}_{p,n}(\mathbf{M} + w\mathbf{\Lambda}, \mathbf{\Sigma}, \mathbf{\Psi})$. Consequently, the property of the matrix variate normal distribution leads to $\boldsymbol{\mathcal{Y}}\mathbf{\Psi}^{-\frac{1}{2}}|W = w \sim \mathcal{N}_{p,n}(\mathbf{M}\mathbf{\Psi}^{-\frac{1}{2}} + w\mathbf{\Lambda}\mathbf{\Psi}^{-\frac{1}{2}}, \mathbf{\Sigma}, \boldsymbol{I})$. Now, by definition 2.1 of [23], we have

$$\boldsymbol{\mathcal{Y}}\mathbf{\Psi}^{-\frac{1}{2}}\mathbf{A}\mathbf{\Psi}^{-\frac{1}{2}}\boldsymbol{\mathcal{Y}}|W = w \sim \mathcal{Q}_p(\mathbf{A}, \mathbf{M}\mathbf{\Psi}^{-\frac{1}{2}} + w\mathbf{\Lambda}\mathbf{\Psi}^{-\frac{1}{2}}, \mathbf{\Sigma}).$$

On the other hand, through theorem 2.2 of [23], we have

$$\sum_{j=1}^{r} \delta_j \boldsymbol{B}_j \big| W = w \sim \mathcal{Q}_p(\mathbf{A}, \mathbf{M}\mathbf{\Psi}^{-\frac{1}{2}} + w\mathbf{\Lambda}\mathbf{\Psi}^{-\frac{1}{2}}, \mathbf{\Sigma}).$$

Therefore, the random matrices $\mathcal{Q}$ and $\boldsymbol{B}$ have identical distributions. "

4) *Parameter estimation: if possible, please provide appropriate reference notes.*

Response:

The reference [18] is acknowledged.

5) *Simulation studies: Is it possible to provide more experimental examples? More examples will reduce the impact of specific data.*

Response:

A new simulation study has been added. Please see "Example 2" in the main text.

5) *Example 1 Model performance: Please explain why such data intervals are used (n=50, 100, 500, 1000, 2000), it is neither geometric progression nor arithmetic progression.*

Response:

We would like to emphasize that the purpose of choosing various sample sizes in this simulation study is to check its effect on the model fitting and parameters accuracy. We checked a geometric increase in the sample size and observed no trend on it.

6) *Fig 1.: Please note if the drawing has been lengthwise stretched.*

Response:

The note has been added. Thanks for raising this point.

7) *Further minor comments directly annotated to the pdf of the paper:*

Response:

We thank the highlighted corrections and suggestions from the reviewer in the pdf. All of the directly annotated comments have been addressed.

References

[18] Dempster AP, Laird NM, Rubin DB. Maximum likelihood from incomplete data via the EM algorithm. *Journal of the royal statistical society Series B (methodological)*. 1977;39(1):1-38.

[23] Singull M, Koski T. On the distribution of matrix quadratic forms. *Communications in Statistics -Theory and Methods*. 2012;41(18):3403-3415.
